# Whole-genome sequencing reveals that variants in the Interleukin 18 Receptor Accessory Protein 3′UTR protect against ALS

**DOI:** 10.1038/s41593-022-01040-6

**Published:** 2022-03-31

**Authors:** Chen Eitan, Aviad Siany, Elad Barkan, Tsviya Olender, Kristel R. van Eijk, Matthieu Moisse, Sali M. K. Farhan, Yehuda M. Danino, Eran Yanowski, Hagai Marmor-Kollet, Natalia Rivkin, Nancy Sarah Yacovzada, Shu-Ting Hung, Johnathan Cooper-Knock, Chien-Hsiung Yu, Cynthia Louis, Seth L. Masters, Kevin P. Kenna, Rick A. A. van der Spek, William Sproviero, Ahmad Al Khleifat, Alfredo Iacoangeli, Aleksey Shatunov, Ashley R. Jones, Yael Elbaz-Alon, Yahel Cohen, Elik Chapnik, Daphna Rothschild, Omer Weissbrod, Gilad Beck, Elena Ainbinder, Shifra Ben-Dor, Sebastian Werneburg, Dorothy P. Schafer, Robert H. Brown, Pamela J. Shaw, Philip Van Damme, Leonard H. van den Berg, Hemali Phatnani, Eran Segal, Justin K. Ichida, Ammar Al-Chalabi, Jan H. Veldink, Project MinE ALS Sequencing Consortium, Johnathan Cooper-Knock, Kevin P. Kenna, Pamela J. Shaw, Philip Van Damme, Leonard H. van den Berg, Ammar Al-Chalabi, Jan H. Veldink, NYGC ALS Consortium, Hemali Phatnani, Eran Hornstein, Eran Hornstein

**Affiliations:** 1 Department of Molecular Genetics, Weizmann Institute of Science, Rehovot, Israel; 2 Department of Molecular Neuroscience, Weizmann Institute of Science, Rehovot, Israel; 3 Department of Computer Science And Applied Math, Weizmann Institute of Science, Rehovot, Israel; 4 Department of Neurology, University Medical Center Utrecht Brain Center, Utrecht University, Utrecht, The Netherlands; 5 KU Leuven - University of Leuven, Department of Neurosciences, Experimental Neurology, Leuven, Belgium; 6 VIB, Center for Brain & Disease Research, Laboratory of Neurobiology, Leuven, Belgium; 7 Analytic and Translational Genetics Unit, Center for Genomic Medicine, Massachusetts General Hospital and Harvard Medical School, Boston, MA, USA; 8 Stanley Center for Psychiatric Research, Broad Institute of MIT and Harvard, Cambridge, MA, USA; 9 Department of Stem Cell Biology and Regenerative Medicine, Keck School of Medicine, University of Southern California, Los Angeles, CA, USA; 10 Eli and Edythe Broad CIRM Center for Regenerative Medicine and Stem Cell Research at USC, Los Angeles, CA, USA; 11 Zilkha Neurogenetic Institute, Keck School of Medicine of the University of Southern California, Los Angeles, CA, USA; 12 Sheffield Institute for Translational Neuroscience (SITraN), University of Sheffield, Sheffield, UK; 13 Inflammation Division, The Walter and Eliza Hall Institute of Medical Research, Parkville, Australia; 14 Department of Medical Biology, University of Melbourne, Parkville, Australia; 15 King’s College London, Maurice Wohl Clinical Neuroscience Institute, Institute of Psychiatry, Psychology & Neuroscience, De Crespigny Park, London, United Kingdom; 16 Department of Developmental Biology, Stanford University, Stanford, CA, USA; 17 Department of Genetics, Stanford University, Stanford, CA, USA; 18 Department of Life Sciences Core Facilities, Weizmann Institute of Science, Rehovot, Israel; 19 Department of Neurobiology, Brudnick Neuropsychiatric Research Institute, University of Massachusetts Chan Medical School, Worcester, MA, USA; 20 Department of Neurology, University of Massachusetts Medical School, Worcester, MA, USA; 21 University Hospitals Leuven, Department of Neurology, Leuven, Belgium; 22 Center for Genomics of Neurodegenerative Disease, New York Genome Center, New York, USA; 23 King’s College Hospital, Denmark Hill, London, United Kingdom

## Abstract

The noncoding genome is substantially larger than the protein-coding genome but has been largely unexplored by genetic association studies. Here, we performed region-based rare variant association analysis of >25,000 variants in untranslated regions of 6,139 amyotrophic lateral sclerosis (ALS) whole genomes and the whole genomes of 70,403 non-ALS controls. We identified interleukin-18 receptor accessory protein (*IL18RAP*) 3′ untranslated region (3′UTR) variants as significantly enriched in non-ALS genomes and associated with a fivefold reduced risk of developing ALS, and this was replicated in an independent cohort. These variants in the *IL18RAP* 3′UTR reduce mRNA stability and the binding of double-stranded RNA (dsRNA)-binding proteins. Finally, the variants of the *IL18RAP* 3′UTR confer a survival advantage for motor neurons because they dampen neurotoxicity of human induced pluripotent stem cell (iPSC)-derived microglia bearing an ALS-associated expansion in *C9orf72*, and this depends on NF-κB signaling. This study reveals genetic variants that protect against ALS by reducing neuroinflammation and emphasizes the importance of noncoding genetic association studies.

ALS is a fatal neurodegenerative syndrome that primarily affects the human motor neuron system and has a strong genetic predisposing component^1,2^. Thus far, mutations in approximately 25 protein-coding genes have been associated with ALS^1,3–5^. Hexanucleotide repeat expansion in an intronic sequence of the *C9orf72* gene is the most common genetic cause of ALS^6–8^, and enrichment of variants was recently discovered in the *CAV1* enhancer^9^. However, noncoding nucleotide variants in ALS have yet to be systematically explored.

Gene-based rare variant association analysis is a genetics approach that is based on the rationale that different rare variants in the same gene may have a cumulative contribution^10^. Therefore, rare variant analytical approaches allow for the identification of genes containing an excess of rare and presumably functional variation in affected individuals relative to healthy individuals. Although mutations in noncoding regions are expected to be numerous^11,12^ and were recently shown in family-based autism studies^13^, variants in noncoding regions are not routinely included in rare variant association studies. The application of rare variant association analysis to noncoding regulatory regions is constrained by the availability of whole-genome sequencing (WGS) data and the ability to recognize functional variants in noncoding regulatory regions, which is currently far less effective than for protein-coding genes.

Noncoding variation may be of particular relevance to ALS. For example, microRNA (miRNA) dysregulation has been implicated in ALS pathogenesis, and ALS-associated RNA-binding proteins TARDBP/TDP-43 and FUS regulate miRNA biogenesis“ ^14–25^. miR-NAs are endogenous posttranscriptional repressors that silence mRNA expression through sequence complementarity. miRNAs primarily act on 3′UTRs^26^, which are noncoding parts of mRNAs and often regulate degradation and translation^27^.

Here, we identified rare variants in miRNAs and in 3′UTRs of mRNAs and performed collapsed genetic analysis^28^ to test if these regulatory RNAs are associated with ALS. We discovered an enrichment of rare variants in the *IL18RAP* 3′UTR in non-ALS genomes, which are associated with a fivefold reduced risk of developing ALS. That association was replicated in an independent cohort and suggests that the *IL18RAP* 3′UTR variants protect against ALS. Mechanistically, the 3′UTR variants reduce *IL18RAP* mRNA association with RNA-binding proteins, destabilize the mRNA and further downregulate IL18RAP–NF-κB signaling in microglia. Microglia induce motor neuron death in ALS, which is mediated, in part, via triggering the IL-18 (refs. ^29–42^) and NF-κB signaling pathway^29,30,43–53^. Finally, we demonstrate that the variant *IL18RAP* 3′UTR dampens neurotoxicity of human iPSC-derived microglia bearing an ALS-associated expansion in *C9orf72* (C9-ALS microglia) in an NF-κB-dependent manner. Therefore, noncoding variant analysis reveals a genetic and mechanistic link for the IL-18 pathway in ALS and encourages systematic exploration of noncoding regions to uncover genetic mechanisms of disease.

## Results

### Rare variants in the *IL18RAP* 3′UTR are associated with ALS

**Extended Data Fig. 1:**
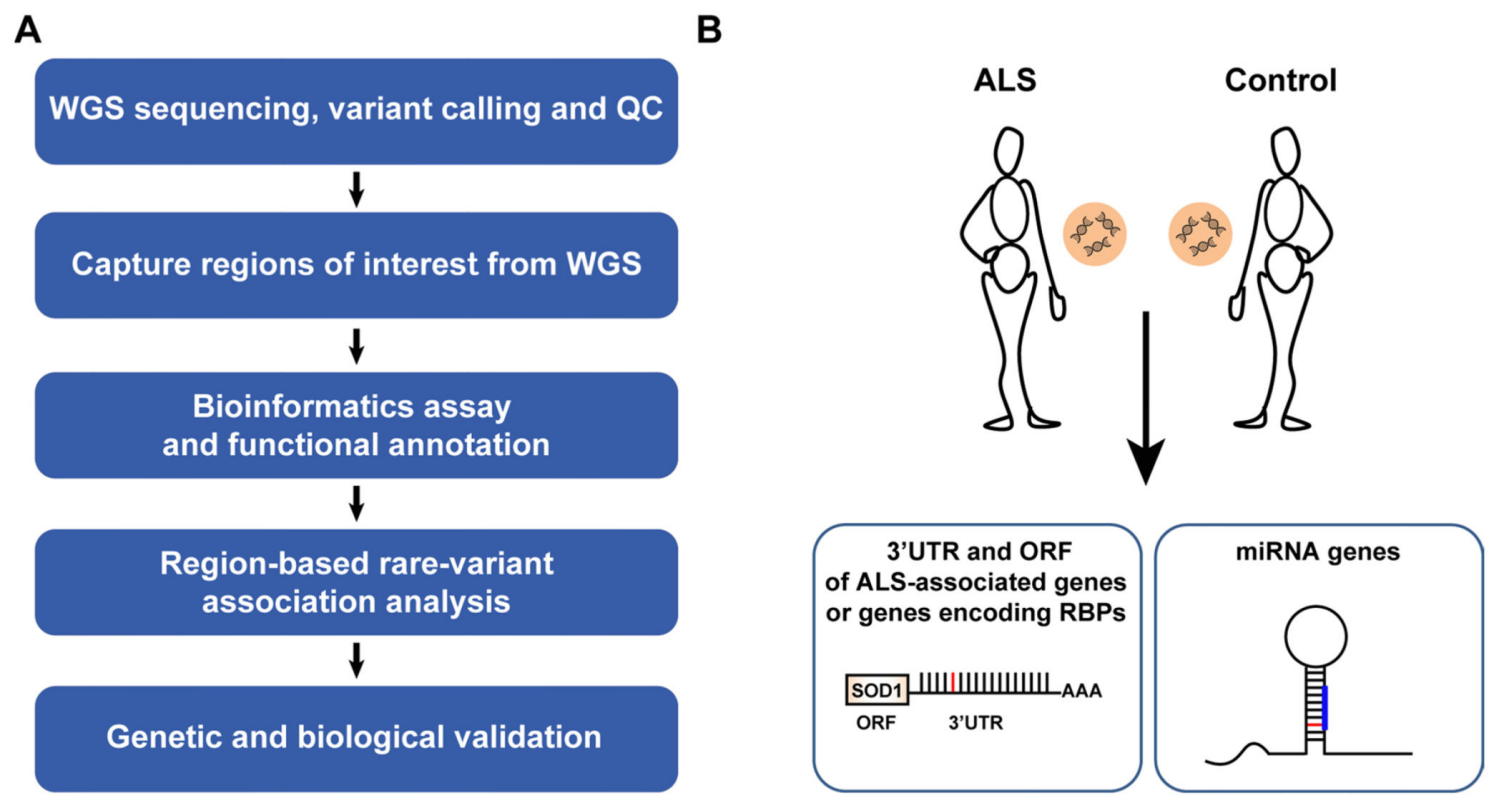
Study design. **(a)** Flow chart of approach for discovery of region-based rare-variants in non-coding genomic regions via association studies and **(b)** diagram depicting regions of interest comprising of 1,750 autosomal human pre-miRNA genes, 295 open reading frames encoding for proteins of interest, and 295 3′UTRs.

To test whether genetic variations in noncoding regulatory regions are associated with ALS, we analyzed regions of interest in WGS data from the Project MinE ALS sequencing consortium^54^ (Extended Data Fig. 1a,b and Supplementary Tables 1 and 2). The discovery cohort consisted of 3,955 individuals with ALS and 1,819 age- and sex-matched healthy individuals, for a total of 5,774 whole genomes from the Netherlands, Belgium, Ireland, Spain, United Kingdom, United States and Turkey (Project MinE Datafreeze 1). We performed a region-based rare variant association test^55^, in which rare genetic variants with minor allele frequencies (MAF) ≤ 0.01 from genomic regions of interest, were binned together to weight their contribution to disease; Region-based rare variant association test was performed on 295 noncoding 3′UTRs of candidate genes that were linked to sporadic ALS via genome-wide association studies^56^ or genes encoding RNA-binding proteins (Supplementary Table 3). In addition, we tested all autosomal human pre-miRNA genes (1,750 pre-miRNAs; miRBase v20 (ref. ^57^)).

**Extended Data Fig. 2:**
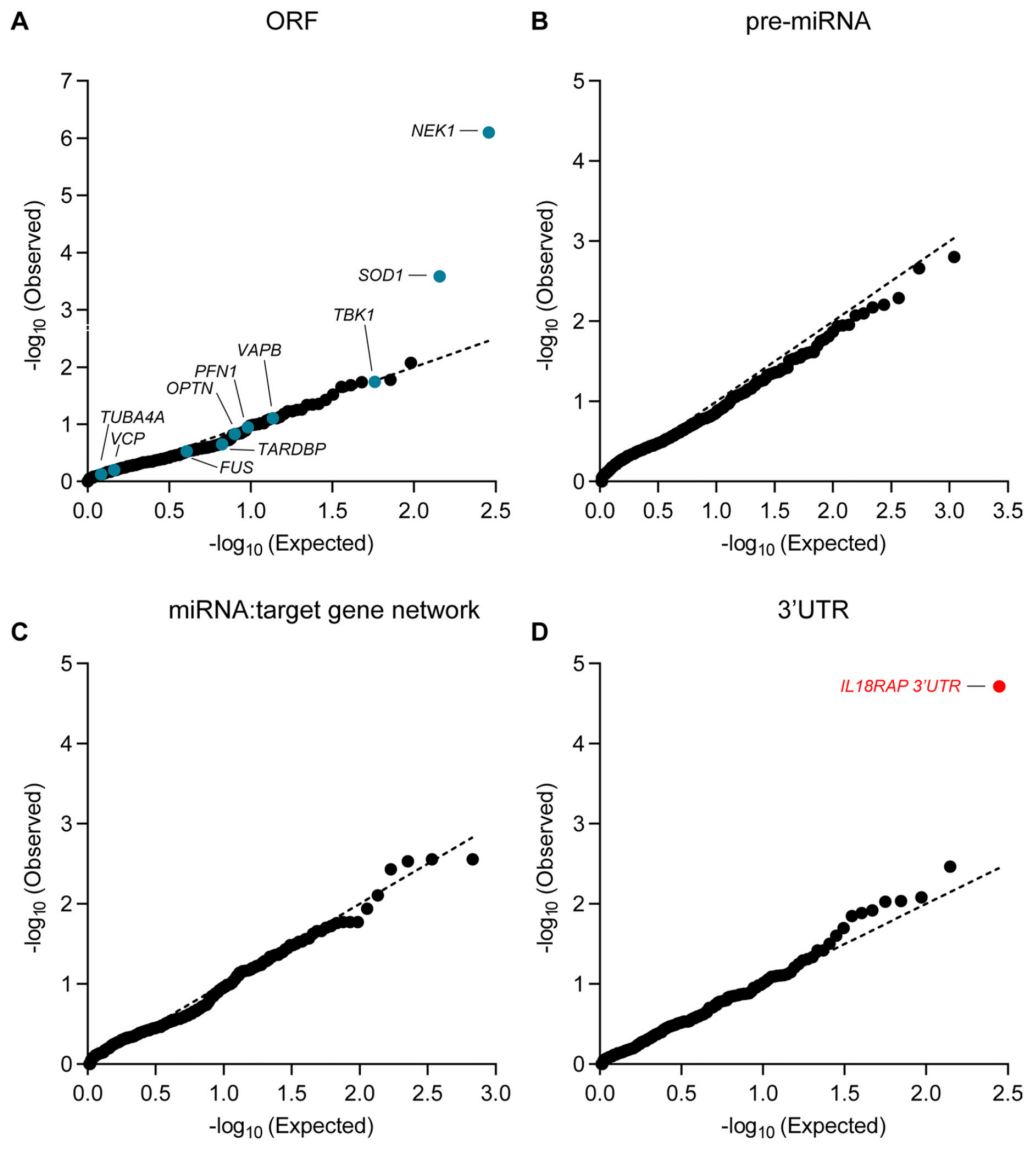
Region-based rare-variant association analyses. **(a-d)** QQ (quantile-quantile) probability plot, of obtained and expected P-values (log scale) gained by region-based rare-variant association analysis of different genomic regions, comprised of **(a)** 295 candidate protein-coding regions listed in Supplementary Table 3. These ORFs encode for ALS-relevant proteins or proteins that are associated with miRNA biogenesis or activity. Variants were depicted if predicted to cause frameshifting, alternative splicing, abnormal stop codon or a deleterious non-synonymous amino acid substitution, in ≥ 3 of 7 independent dbNSFP prediction algorithms (genomic inflation λ = 0.96), **(b)** All known pre-miRNA genes in the human genome (genomic inflation λ = 1.31), **(c)** predicted networks, comprised of aggregated variants detected on a specific mature miRNA sequence and its cognate downstream 3′UTR targets (genomic inflation λ = 1.16), and **(d)** variants in 3′UTRs of the same 295 genes listed in Supplementary Table 3 (genomic inflation λ = 1.08). Data was obtained from 3,955 ALS cases and 1,819 controls (Project MinE). Features positioned on the diagonal line represent results obtained under the null hypothesis. Open-reading frames of 10 known ALS genes (blue). IL18RAP 3′UTR (red). P-values, calculated with SKAT-O.

**Fig. 1:**
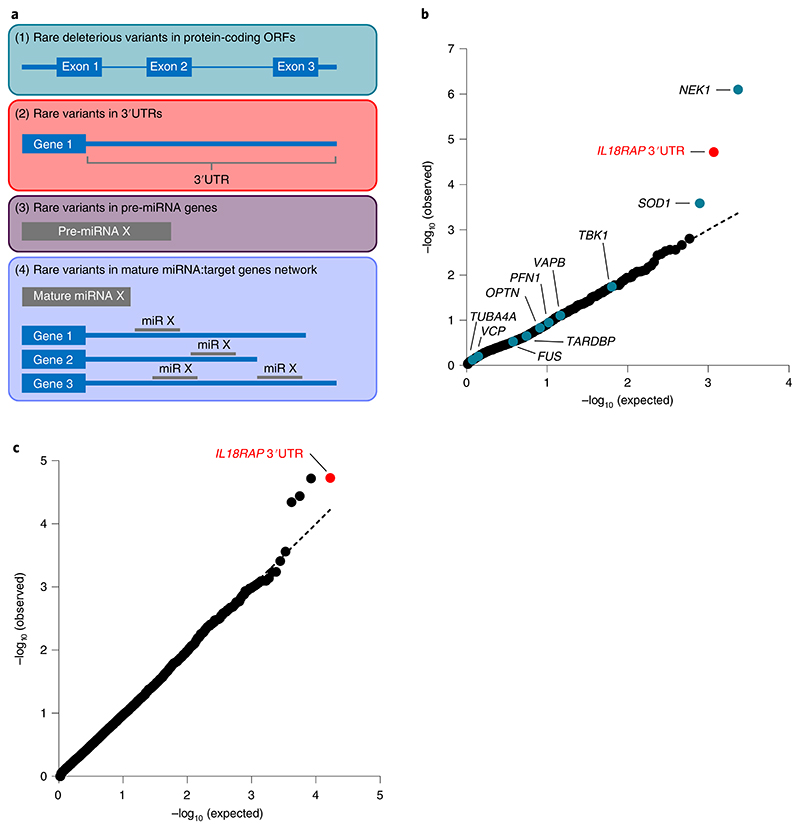
Region-based rare variant association analysis reveals association of the *IL18RAP* 3′UTR with ALS. **a**, Diagram of study design. A collapsed region-based rare variant (MAF ≤ 0.01) association analysis was performed on the following: (1) 295 candidate protein-coding genes (Supplementary Table 3) encoding ALS-relevant proteins or proteins associated with miRNA biogenesis/activity; variants were included if predicted to cause frameshifting, alternative splicing, an abnormal stop codon or a deleterious non-synonymous amino acid substitution in ≥ three of seven independent dbNSFP prediction algorithms; (2) variants in 3′UTRs of the 295 genes (Supplementary Table 3); (3) all known autosomal pre-miRNA genes in the human genome and (4) predicted networks, comprised of aggregated variants detected in a specific mature miRNA sequence and its cognate downstream 3′UTR targets; ORF, open reading frame. **b**, QQ (quantile-quantile) probability plot of obtained and expected *P* values (log scale) gained by region-based rare variant association analysis of all genomic regions described in **a**. Data were obtained from 3,955 individuals with ALS and 1,819 healthy individuals (Project MinE). Features positioned on the diagonal line represent results obtained under the null hypothesis; blue, open reading frames of 10 known ALS genes; red, *IL18RAP* 3′UTR; genomic inflation *λ* = 1.2. **c**, QQ plot of obtained and expected *P* values (log scale) gained by region-based rare variant association analysis for all known human 3′UTRs (RefSeq). The *IL18RAP* 3′UTR (red) is the most significant 3′UTR associated with ALS. *P* values were calculated with SKAT-O (genomic inflation *λ =* 0.97).

As a positive control, we also performed an association analysis of rare variants in the open reading frames of these 295 genes. For the proteins, we called variants that are predicted to cause frameshifting, alternative splicing, an abnormal stop codon or a deleterious non-synonymous amino acid substitution that was detected in ≥three of seven independent dbNSFP prediction algorithms^58^ (Fig. 1a and Supplementary Table 3). In total, 30,721 rare qualifying variants were identified (Supplementary Table 4). Optimized sequence kernel association test (SKAT-O)^55^ identified a significant excess of deleterious minor alleles in the ALS genes *NEK1* (127 individuals with ALS and 19 healthy individuals (3.21% and 1.04%): *P* = 8 × 10^−7^; *P*_corrected_ = 2.3 × 10^−4^), comparable with a reported prevalence of 3% (ref. ^59^), and *SOD1* (36 individuals with ALS (0.91%) and 0 healthy individuals: *P* = 2.6 × 10^−4^ and *P*_corrected_ = 3.73 × 10^−2^)^60^, which is below the reported 2% prevalence^3,61^ (Fig. 1b, Extended Data Fig. 2a and Supplementary Table 5). Other known ALS genes did not reach statistical significance (Supplementary Table 3), consistent with reported statistical power limitations of Project MinE WGS data in assessing the burden of rare variants^62^. Our analysis did not consider the *C9orf72* hexanucleotide (GGGGCC) repeat expansion region.

The rare variant association test did not identify a disease association for any of the autosomal pre-miRNAs in the human genome, nor for any of the predicted genetic networks based on variants aggregated over specific mature miRNAs and their cognate downstream 3′UTR targets. This may be because the small size of miRNA genes makes genetic aggregation studies particularly challenging (Extended Data Fig. 2b,c).

When we tested the association of rare variants in 3′UTRs, the strongest association found was for the 3′UTR of *IL18RAP* (also known as AcPL/CD218b/IL-18R-β; Fig. 1b, Extended Data Fig. 2d and Supplementary Table 5). This association was higher than expected at random (*P* = 1.93 × 10^−5^; *P*_corrected_ = 5.41 × 10^−3^) and from the association gained for all protein-coding ALS genes in this cohort, with the exception of *NEK1*. Notably, the signal was more prevalent in healthy individuals (12/1,819, 0.66%) than in individuals with ALS (6/3,955, 0.15%), indicating that these variants might act as protective variants against ALS.

**Extended Data Fig. 3:**
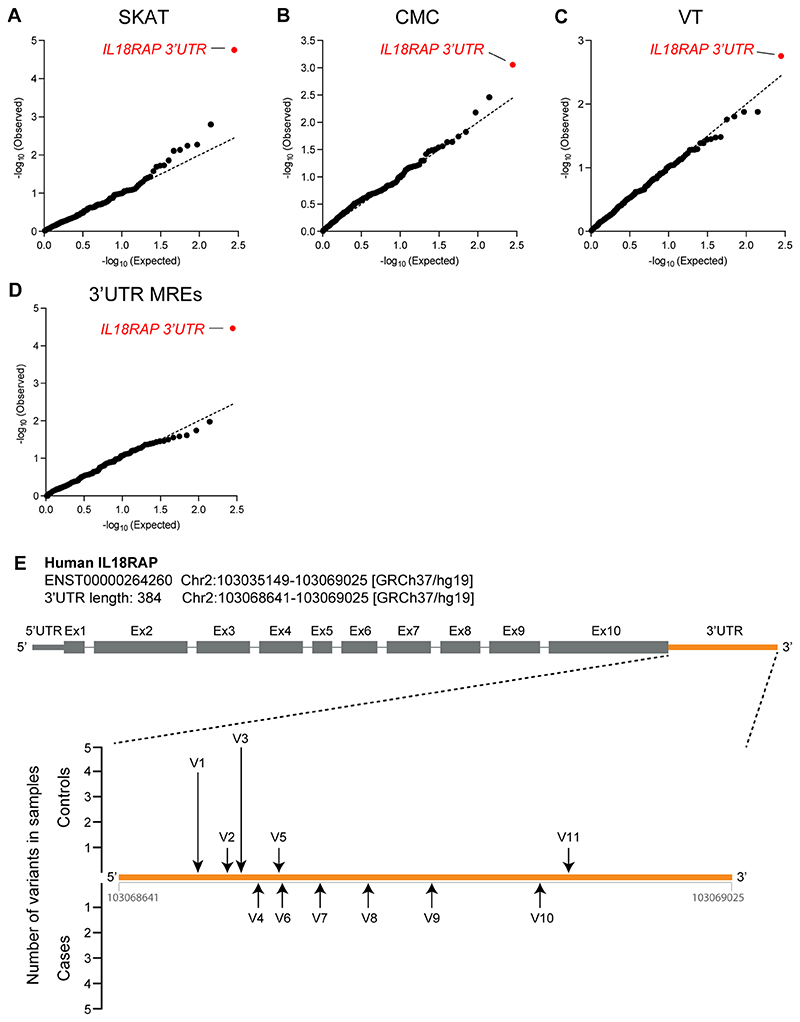
3′UTR-based rare-variant association analysis, using different algorithms, and illustration of rare variants identified in the IL18RAP 3′UTR. **(a-d)** QQ plot of obtained and expected P-values (log scale) gained by region-based rare-variant association analysis of genomic regions comprised of 295 3′UTRs listed in Supplementary Table 3, in the Project MinE cohort (3,955 ALS cases and 1,819 non-ALS controls). Features positioned on the diagonal line represent results obtained under the null hypothesis. IL18RAP 3′UTR (red) is the most significant 3′UTR associated with ALS using different algorithms: **(a)** Sequence Kernel Association Test, SKAT (genomic inflation λ = 1.02), **(b)** Combined Multivariate and Collapsing, CMC (genomic inflation λ = 1.34), **(c)** Variable Threshold with permutation analysis, VT (genomic inflation λ = 1.03). **(d)** IL18RAP 3′UTR also ranked as the top hit when aggregating variants abrogating or gaining miRNA recognition elements (MREs) in 3′UTRs (genomic inflation λ = 1.04). **(e)** Schematic of the IL18RAP transcript and 3′UTR (5′ to 3′) showing the number of control (upper) or ALS (lower) samples in which variants (black arrow) were identified in the Project MinE discovery cohort (Supplementary Table 6).

The *IL18RAP* 3′UTR was also ranked as the top hit by three other algorithms: the sequence kernel association test (SKAT; *P* = 1.77 × 10^−5^; permutated *P* < 10^−4^), the combined multivariate and collapsing (CMC) analysis (*P* = 8.78 × 10^−4^) or variable threshold (VT) with permutation analysis (permutated *P* = 1.75 × 10^−3^), suggesting that the association does not depend on a particular statistical genetics method (Extended Data Fig. 3a–c). Furthermore, when we tested the association of rare variants in miRNA recognition elements in 3′UTRs (variants that are potentially either abrogating conserved miRNA-binding sites or creating new miRNA-binding sites in 3′UTRs), the strongest association was also gained for the 3′UTR of *IL18RAP* (SKAT-O, *P* = 3.42 × 10^−5^; Extended Data Fig. 3d and Methods). A diagram of variants in the *IL18RAP* 3′UTR is presented in Extended Data Fig. 3e, and a description of *IL18RAP* 3′UTR variants is in Supplementary Table 6. The top 10 principal components (PCs) of common variant-based ancestry information and sex were included as covariates in the SKAT-O, SKAT, CMC and VT analyses.

In addition, genome-wide analysis of all known human 3′UTRs (16,809 3′UTRs from RefSeq^63^) identified the *IL18RAP* 3′UTR as the most significant 3′UTR associated with ALS in the Project MinE cohort (Fig. 1c).

Finally, we tested if different functional genetic classes were enriched overall for ALS risk/protection variants by testing the association of rare variants in all genes pooled together. SKAT-O signal for open reading frames of 295 proteins, the 3′UTRs of the same 295 genes, all autosomal pre-miRNA genes (miRBase v20 (ref. ^57^)) or networks composed of all miRNA genes and their cognate set of downstream targets (TargetScan) were all not significant following correction to these four hypotheses (adjusted *P* values of 0.096, 0.59, 0.16 and 0.77, respectively). Therefore, results from these rare variant association tests do not implicate any of the functional classes of genomic elements in ALS risk.

### *IL18RAP* 3′UTR variants reduce the odds of suffering from ALS

**Fig. 2:**
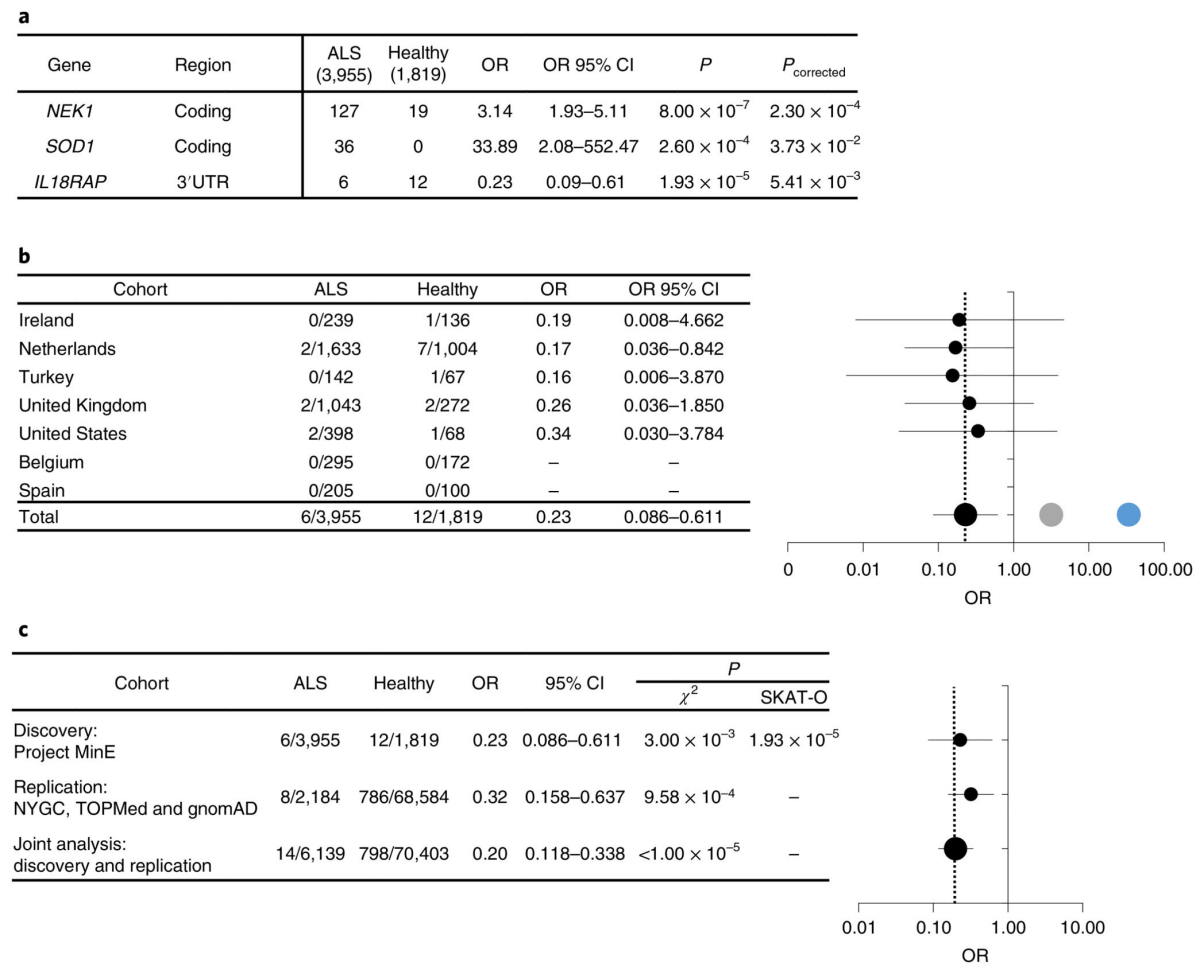
Odds of ALS are reduced with rare variants in the *IL18RAP* 3′UTR. **a**, OR estimates with 95% confidence intervals (CI) for *NEK1* (coding), *SOD1* (coding) and *IL18RAP* (3′UTR). SKAT-O *P* values are corrected for false discovery rate (FDR). **b**, Stratification of data pertaining to the *IL18RAP* 3′UTR in seven geographically based sporadic ALS subcohorts and forest plot (OR on log scale with whiskers for 95% CI). *NEK1* (gray) and *SOD1* (blue) signals are from combined data of all cohorts. The vertical dotted line denotes OR = 0.23. **c**, Stratification of *IL18RAP* 3′UTR variant data across discovery and replication cohorts and joint analysis thereof; forest plot, OR on log scale with whiskers for 95% CI. The vertical dotted line denotes OR = 0.2. *P* values were calculated with SKAT-O or a chi-squared test with Yate’s correction.

Because the number of ALS genomes was ~2.17-fold larger than the number of control genomes, the data depict a 4.35-fold enrichment in the abundance of variants in healthy individuals over individuals with ALS. *IL18RAP* 3′UTR potentially protective variants reduced the disease odds ratio (OR) by fivefold (OR = 0.23; Fig. 2a) and were consistent across independent population strata (Fig. 2b), whereas *NEK1* and *SOD1* increased the disease OR (OR = 3.14 and 33.89, respectively; Fig. 2a).

To determine if the rare *IL18RAP* 3′UTR variants are depleted in another ALS cohort, we performed independent replication studies. Similar results for rare *IL18RAP* 3′UTR variants were reproduced in the New York Genome Center (NYGC) ALS Consortium cohort (2,184 ALS genomes), which was studied against: (1) 263 non-neurological controls from the NYGC, (2) 62,784 non-ALS genomes from the National Heart, Lung and Blood Institute’s (NHLBI’s) Trans-Omics for Precision Medicine (TOPMed) and (3) 5,537 non-ALS genomes from Genome Aggregation Database (gnomAD). This replication effort yielded a joint analysis *P* value of 9.58 × 10^−4^ (*χ*^2^ with Yate’s correction; OR = 0.32; 95% CI: 0.16–0.64; Fig. 2c and Supplementary Table 7). Combining this cohort with our discovery cohort from Project MinE yielded a superior joint *P* value of <1.00 × 10^−5^ (*χ*^2^ with Yate’s correction; OR = 0.20; 95% CI: 0.12–0.34; Fig. 2c). A meta-analysis of Project MinE Datafreeze 1 and 2 (ref. ^5^), which consisted of 5,185 individuals with ALS and 2,262 age- and sex-matched healthy individuals, reproduced the initial signal (*P* = 7.6 × 10^−4^).

Together, *IL18RAP* 3′UTR sequence variants are associated with a nearly fivefold lower risk of suffering from ALS, although it did not reach conventional exome-wide multiplicity-adjusted significance threshold (*α* ≈ 2.6 × 10^−6^ (ref. ^10^)) in our study.

To investigate the source of the signal in the *IL18RAP* 3′UTR in a post hoc analysis, we divided the 11 variant nucleotides into two subsets of either 9 singleton variants (9 variants/3 healthy individuals/6 individuals with ALS) or 2 variants that were identified solely in healthy individuals (2 variants/9 healthy individuals/0 individuals with ALS). While the signal of the nine singleton variants was not statistically significant, analysis of the two control variants, which were identified in multiple samples, derived an improved significance compared to the original signal (SKAT-O *P* value of 4.36 × 10^−6^). Thus, these two rare variants (V1, chr2:103068691 C > T; V3, chr2:103068718 G > A) are likely central in generating the genetic association signal in the *IL18RAP* 3′UTR.

**Extended Data Fig. 4:**
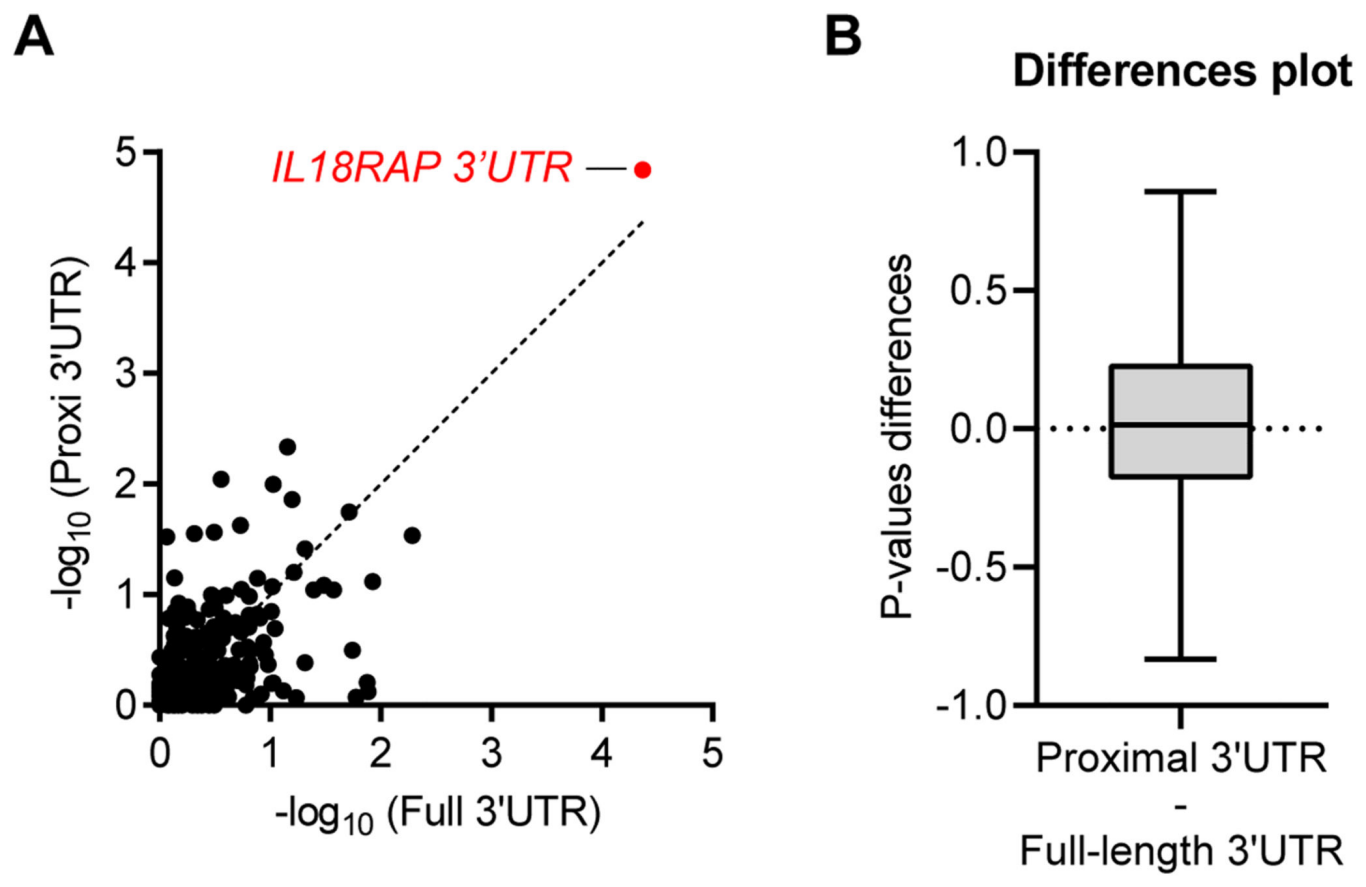
Restricting rare-variant association analysis to the proximal part of 3′UTRs does not improve the association signal. **(a)** Scatter plot with SKAT-O P-values (log scale) calculated for region-based rare-variant association analysis of the full 3′UTRs on the x-axis versus the 3′UTRs proximal quadrant on the y-axis, for the 295 3′UTRs listed in Supplementary Table 3, in the Project MinE cohort (3,955 ALS cases and 1,819 non-ALS controls) (Pearson correlation coefficient (r=0.61) and P-value ****< 0.0001). The 45-degree diagonal line represents a perfect correlation of r=1. IL18RAP 3′UTR (red). **(b)** A Difference plot showing the difference between the two P-value measurements (3′UTRs proximal quadrant minus the full 3′UTRs, for the cohort of N=295 3′UTRs). The bias (difference between means) is only 0.03. Overall the P-values gained from the 3′UTRs proximal quadrant were comparable to that of the full 3′UTRs in the cohort of 295 3′UTRs. For box plot, the median is indicated by the central line, upper and lower quartiles are indicated by the box, and maximum/minimum values are indicated by the whiskers (Wilcoxon matched-pairs P-value > 0.05, Cohen’s d effect size = 0.1). Hence, the apparent spatial distribution of variants in IL18RAP 3′UTR seems to be a particular case, rather than part of a global pattern.

Because of the enrichment of V1 and V3 at the proximal (5′) side of the *IL18RAP* 3′UTR, we tested if restricting rare variant association analysis to the 5′ end of the 3′UTRs might boost the association signal. However, the *P* values gained from the 3′UTRs proximal quadrants were comparable to those of full 3′UTRs in the cohort of 295 3′UTRs (Wilcoxon matched pairs *P* value of >0.05, Cohen’s *d* effect size = 0.1; Extended Data Fig. 4a,b), suggesting that the apparent spatial distribution of variants in the case of the *IL18RAP* 3′UTR is a particular case rather than part of a global pattern.

### Variant *IL18RAP* 3′UTR is associated with reduced IL-18–NF-κB signaling

To determine the functional impact of the *IL18RAP* 3′UTR variants, we analyzed IL18RAP expression in lymphoblastoid cell lines (LCLs) from the UK MNDA DNA Bank^64^ that were derived from 12 different individuals: 4 healthy individuals (without ALS) carrying the canonical *IL18RAP* 3′UTR sequence (control; canonical *IL18RAP* 3′UTR), 4 individuals with sporadic ALS carrying the canonical *IL18RAP* 3′UTR sequence (sALS; canonical *IL18RAP* 3′UTR), 2 healthy individuals carrying a variant form of the *IL18RAP* 3′UTR (control; variant *IL18RAP* 3′UTR) and 2 individuals with sporadic ALS carrying a variant form of the *IL18RAP* 3′UTR (sALS; variant *IL18RAP* 3′UTR; see Supplementary Table 8 for list of variants).

**Extended Data Fig. 5:**
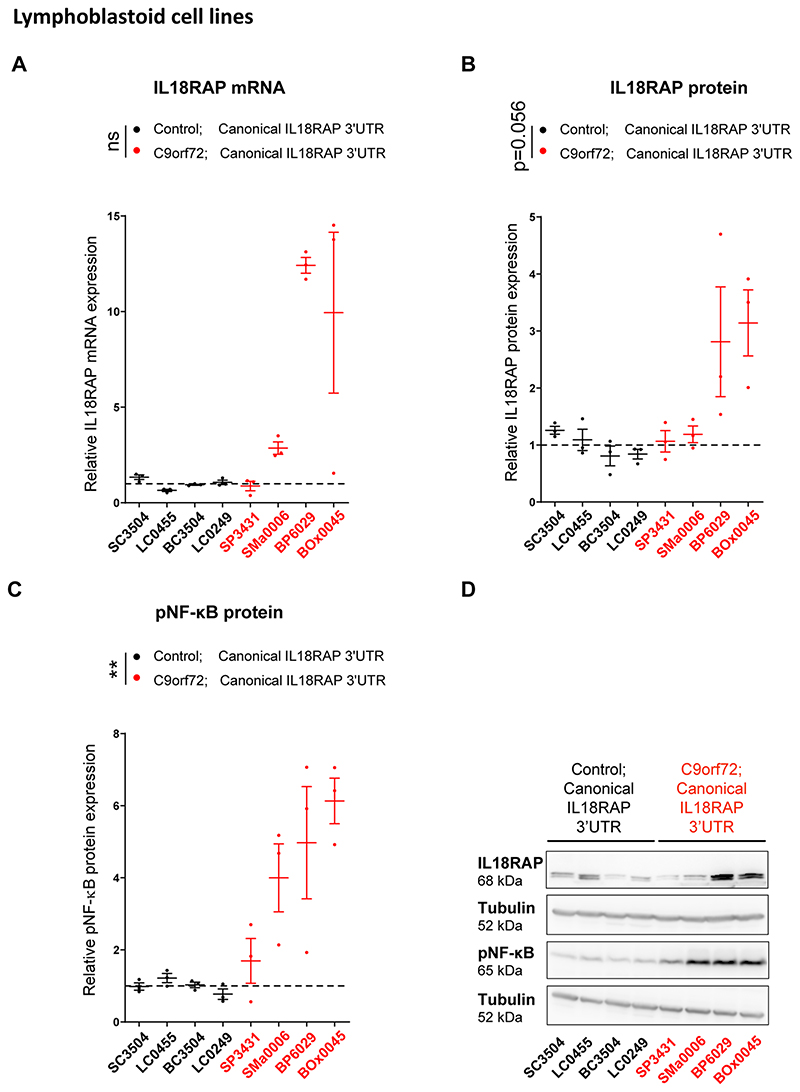
IL18RAP and p-NF-κB expression is elevated in lymphoblastoid cells from patients with the C9orf72 repeat expansion. **(a)** IL18RAP mRNA expression (qPCR normalized to IPO8 mRNA levels) and **(b)** IL18RAP or **(c)** p-NF-κB protein expression (Western blots, normalized to Tubulin). Extracts from eight different human lymphoblastoid cell lines (listed in Supplementary Table 8): Four lines of healthy individuals (without ALS) carrying the canonical IL18RAP 3′UTR sequence (Control; Canonical IL18RAP 3′UTR, black) and four *C9orf72* ALS patients carrying the canonical IL18RAP 3′UTR sequence (*C9orf72;* Canonical IL18RAP 3′UTR, red). **(d)** Representative blots processed with anti-IL18RAP, anti p-NF-κB and anti-Tubulin antibodies. Mann-Whitney test (A) or one-sided student’s t-test with Welch’s correction on log-transformed data (P = 0.056 for panel B; P = 0.0065 for panel C), was conducted based on the mean value of three independent passages for each of the eight human lymphoblastoid cell lines (Source Data Extended Data Fig. 5). Scatter dot plot with mean and SEM. **P<0.01.

**Extended Data Fig. 6:**
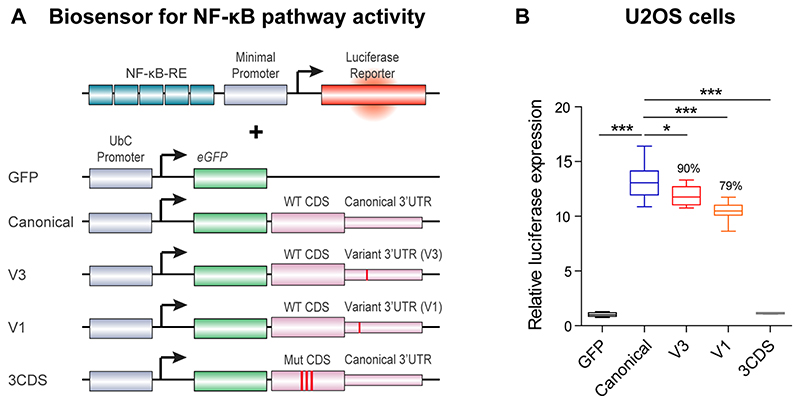
IL18RAP 3′UTR variants attenuate IL-18 - NF-κB signaling in U2OS cells. Diagram **(a)** and quantification **(b)** of NF-κB reporter assays in human U2OS cell line. To determine the ability of the IL18RAP variants V3 and V1 to induce NF-κB activity, U2OS cells were co-transfected with different IL18RAP coding region (CDS) and 3′UTR constructs (GFP, Canonical, V3, V1, n=9; 3CDS, *n*=4), along with an NF-κB activity reporter that drives luciferase (Luc2P) transcription via five copies of the NF-κB response element. NF-κB signaling was induced by adding human recombinant IL-18 to the medium. Variants V3 and V1 of the IL18RAP 3′UTR reduced NF-κB activity by ~10% and ~21%, respectively, relative to the WT IL18RAP 3′UTR. GFP vector and a dominant-negative coding mutant E210A-Y212A-Y214A CDS + WT 3′UTR (3CDS)^31^, served as controls. Luciferase expression was normalized to transfected U2OS cells that were not induced with human recombinant IL-18. One-way ANOVA followed by Dunnett’s multiple comparison test was performed on square root-transformed data. For box plots, the median is indicated by the central line, upper and lower quartiles are indicated by the box, and maximum/minimum values are indicated by the whiskers. * P<0.05; *** P<0.001. The experiment was repeated independently three times with similar results.

**Fig. 3:**
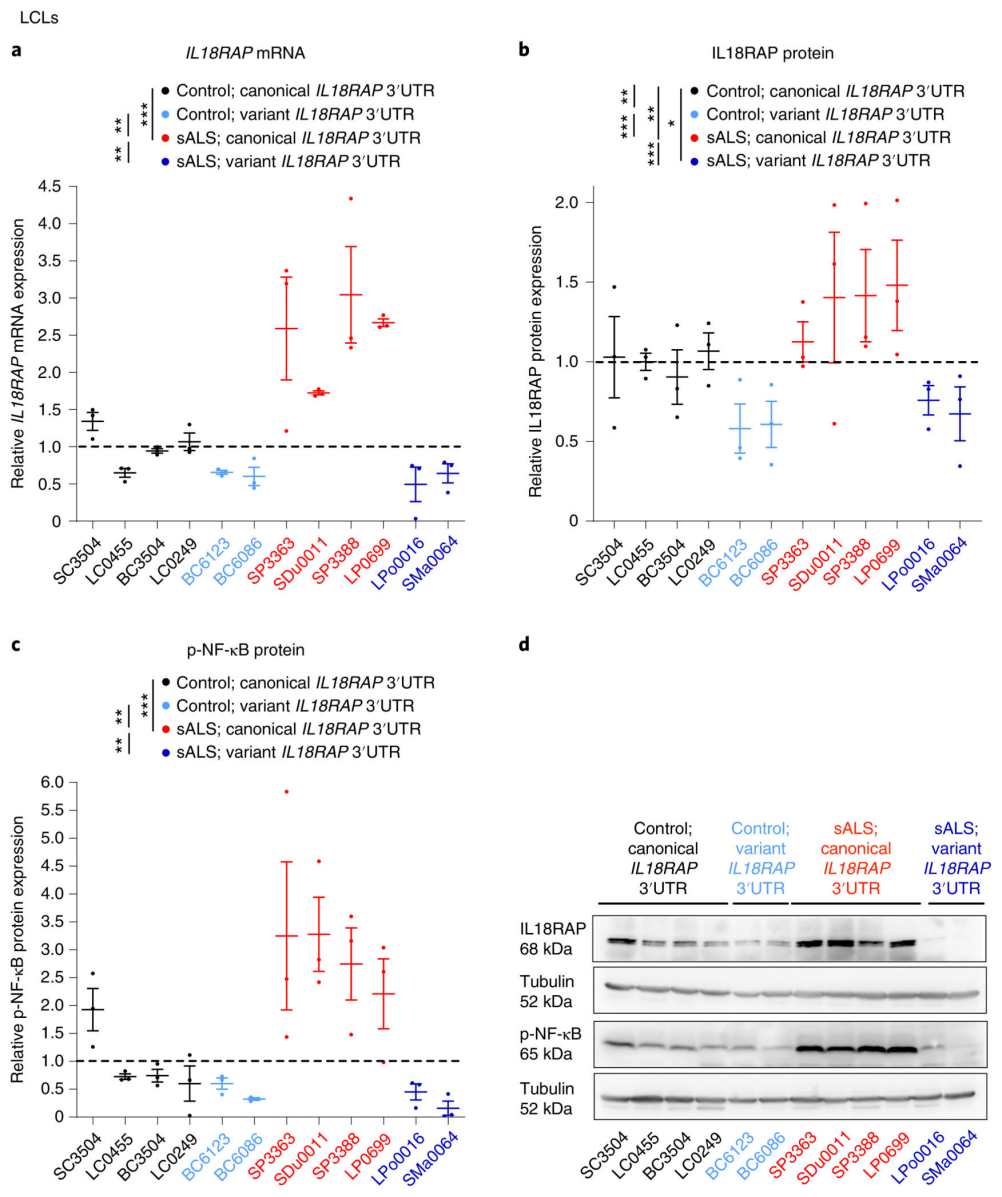
*IL18RAP* 3′UTR variants correlate with attenuated IL-18-NF-κB signaling in human lymphoblastoid cells. **a-c**, *IL18RAP* mRNA expression (quantitative real-time PCR (qPCR) normalized to *IPO8* mRNA levels) (**a**) and IL18RAP (**b**) or p-NF-κB (**c**) protein expression (western blots normalized to tubulin). The scatter dot plot shows mean and s.e.m. values. d, Representative blots processed with anti-IL18RAP, anti-p-NF-κB and anti-tubulin. Extracts from 12 different human LCLs (listed in Supplementary Table 8): 4 lines from healthy individuals (without ALS) carrying the canonical *IL18RAP* 3′UTR sequence (control; canonical *IL18RAP* 3′UTR; black), 4 individuals with sporadic ALS carrying the canonical *IL18RAP* 3′UTR sequence (sALS; canonical *IL18RAP* 3′UTR; red), 2 healthy individuals carrying a variant form of the *IL18RAP* 3′UTR (control; variant *IL18RAP* 3′UTR; light blue) and 2 individuals with sporadic ALS carrying a variant form of the *IL18RAP* 3′UTR (sALS; variant *IL18RAP* 3′UTR; navy blue). A one-way analysis of variance (ANOVA) followed by a Newman–Keuls multiple comparisons test was conducted based on the mean value of three independent passages for each of the 12 human LCLs; *P < 0.05; **P < 0.01; ***P < 0.001.

ALS-derived LCLs carrying the canonical *IL18RAP* 3′UTR sequence expressed higher levels of IL18RAP (Fig. 3a,b). In addition, LCLs from both healthy individuals and individuals with ALS harboring *IL18RAP* 3′UTR variants significantly down-regulated IL18RAP mRNA and protein expression (Fig. 3a,b). Phosphorylation of NF-κB (p-NF-κB), an established intracellular effector downstream of IL-18 signaling, was similarly higher in the ALS LCLs with canonical *IL18RAP* 3′UTR and also significantly reduced in control and ALS LCLs, harboring *IL18RAP* variants (Fig. 3c,d). Consistent results were obtained with *C9orf72* hexanucleotide expansion ALS LCLs (Extended Data Fig. 5). Accordingly, variants of the *IL18RAP* 3′UTR reduced NF-κB activity relative to the canonical 3′UTR in an NF-κB reporter assay in U2OS cells (Extended Data Fig. 6). Therefore, variant forms of the *IL18RAP* 3′UTR correlate with reduced expression of endogenous IL18RAP and reduced NF-κB signaling.

### Variant 3′UTR destabilizes *IL18RAP* mRNA in human microglia

**Fig. 4:**
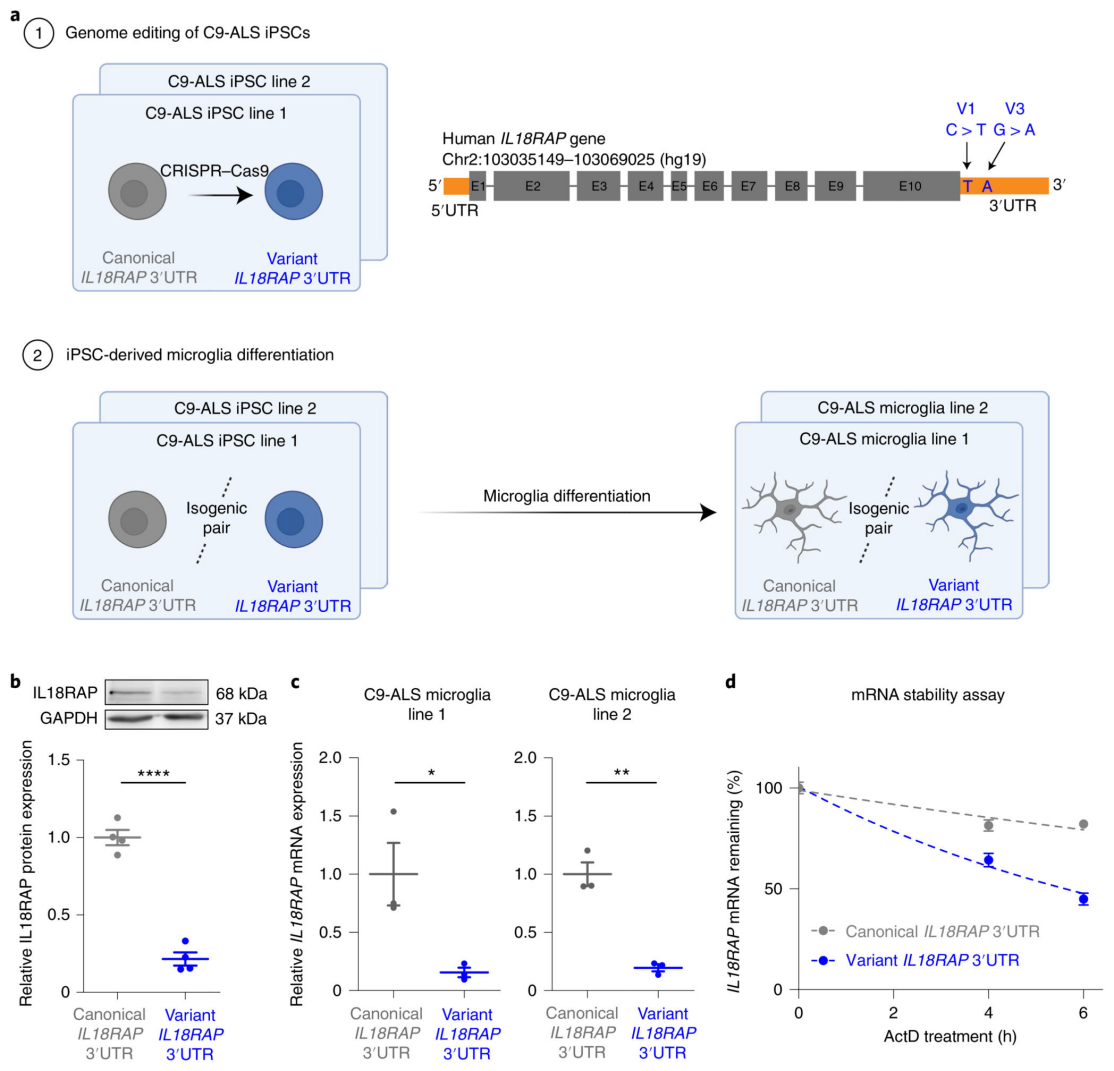
*IL18RAP* 3′UTR variants destabilize *IL18RAP* mRNA in CRISPR-edited isogenic iPSC-derived microglia with a *C9orf72* genetic background. **a**, Diagram of experimental design. (1) Genome editing with CRISPR–Cas9 of point mutations that recapitulate the most prevalent variants (chr2:103068691 C > T (V1) and chr2:103068718 G > A (V3)) in the *IL18RAP* 3′UTR sequence in human iPSCs donated by individuals with a *C9orf72* repeat expansion^65^ (NINDS/Coriell code ND10689, ND12099; Supplementary Table 8). The two independent isogenic pairs of cell lines, both carry the *C9orf72* repeat expansion and vary only by the presence of the canonical or variant *IL18RAP* 3′UTR. (2) The four *IL18RAP* 3′UTR lines (two isogenic pairs) were differentiated into human microglia^67^. **b**,**c**, Dot plots of IL18RAP protein levels (by western blot analysis, normalized to GAPDH; *N* = 3) (**b**) and mRNA expression (by qPCR normalized to *IPO8* mRNA, *N* = 3; C9-ALS microglia line 1, *P* = 0.036; C9-ALS microglia line 2, *P* = 0.0016) (**c**) in differentiated human microglia. **d**, *IL18RAP* mRNA degradation rate studied in human isogenic microglia at 0, 4 and 6 h after introduction of a transcriptional block with actinomycin D (ActD; 7.5 μg ml^−1^; Sigma-Aldrich, A9415; qPCR normalized to the average of *IPO8* and *GAPDH* mRNA expression; *n* = 4 independent wells per time point with two technical duplicates). Variant 3′UTR destabilizes the *IL18RAP* mRNA, relative to the canonical sequence. Scatter dot plots show mean and s.e.m. values; two-sided *t*-test; *P < 0.05; **P < 0.01; ****P < 0.0001.

To further establish the functional relevance of the *IL18RAP* 3′UTR variants, we edited the genome of human iPSCs donated by individuals with ALS with a *C9orf72* repeat expansion^65^ (National Institute of Neurological Disorders and Stroke (NINDS)/Coriell code ND10689, ND12099; Supplementary Table 8), to include two point mutations that recapitulate the most prevalent variants (chr2:103068691 C > T (V1) and chr2:103068718 G > A (V3)) in the *IL18RAP* 3′UTR sequence (Fig. 4a). The resulting isogenic pair lines all carry a *C9orf72* repeat expansion and vary by only the presence of the canonical or variant *IL18RAP* 3′UTR.

**Extended Data Fig. 7:**
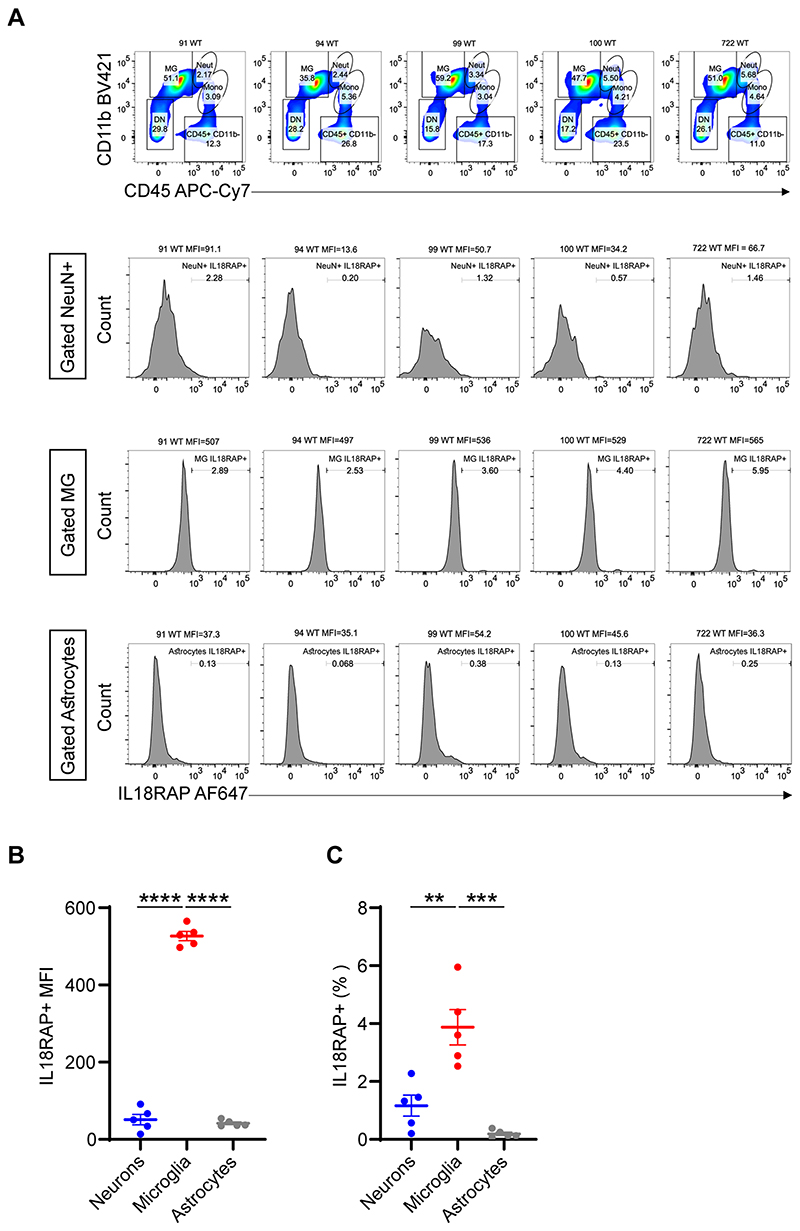
IL18RAP is mainly expressed on mouse microglia cells. **(a-c)** Flow cytometry was used to characterize IL18RAP expression levels in dissociated wild-type mouse cortex cells. The expression of IL-18RAP (IL-18Rβ) was expressed as Mean Fluorescence Intensity (MFI) and % frequency after gating for the following cell types: immune cells (CD45hi), microglia (MG: CD45int CD11hi), neurons (CD45-CD11b-NeuN+), and astrocytes (CD45-CD11b-GFAP+). FACS analysis reveals that IL18RAP is mainly expressed on microglia cells. A scatter dot plot with mean and SEM values for the median fluorescence intensity (MFI) and percentage of IL18RAP+ cells is shown. One-way ANOVA followed by Tukey’s multiple comparison test. ** P<0.01, *** P<0.001, **** P<0.0001.

**Extended Data Fig. 8:**
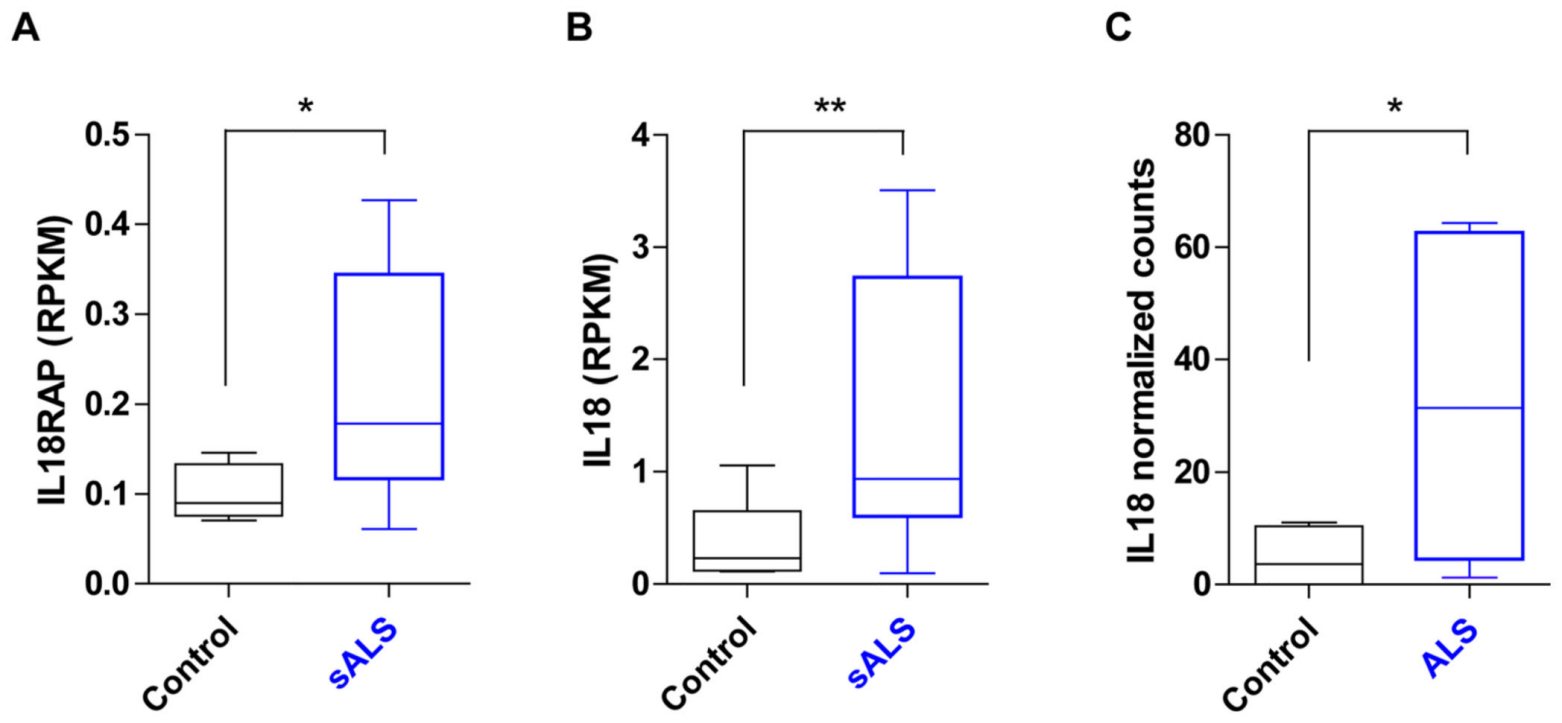
Evaluation of IL18RAP and IL-18 mRNA expression in motor neurons of patients with ALS. **(a-b)** mRNA expression of IL18RAP **(a)** and IL-18 **(b)**, as reads per kilobase million (RPKM), from NGS study of laser capture microdissection–enriched surviving motor neurons from lumbar spinal cords of patients with sALS with rostral onset and caudal progression (n = 12) and non-neurodegeneration controls (n = 9^112^; GSE76220). Two-sided Student’s t test with Welch’s correction on log-transformed data (P = 0.0138 for panel A; P = 0.0056 for panel B). **(c)** IL-18 mRNA expression, as log2-normalized counts, from NGS study of induced ALS motor neurons (n = 4 different donors in duplicates) or non-neurodegeneration controls (n=3 different donors in duplicates^113^; DESeq analysis, P = 0.0417). For box plots, the median is indicated by the central line, upper and lower quartiles are indicated by the box, and maximum/minimum values are indicated by the whiskers. *P < 0.05; **P < 0.01.

We explored the receptive cell type involved in IL-18R signaling by profiling dissociated mouse brain cells, namely, neurons, microglia and astrocytes. Fluorescence cytometric gating on CD11b^+^ and CD45^+^ and IL18RAP (CD218b) revealed that IL18RAP is mainly expressed on microglia cells (Extended Data Fig. 7a–c). Although IL-18 and IL18RAP expression increases in ALS motor neurons (Extended Data Fig. 8a–c), our observations are consistent with the accepted notion that the role of IL-18 and other cytokines in disease heavily rests on a chronic inflammatory state established particularly by microglia^66^.

**Extended Data Fig. 9:**
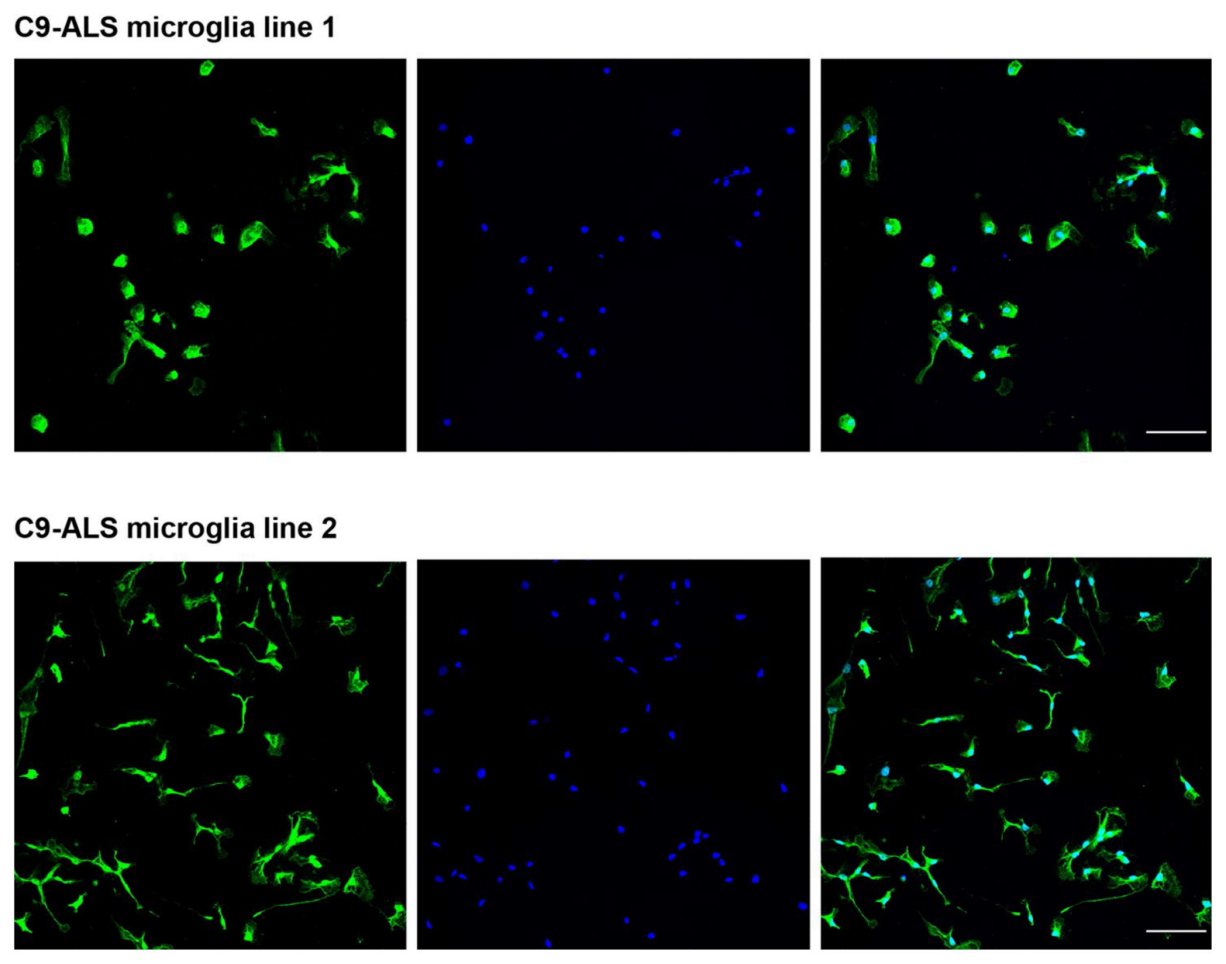
iPSC-derived microglia express the microglial-specific marker, TMEM119. Immunofluorescence staining of TMEM119 (green) and DAPI (blue), in two different C9orf72 iPSC-derived progenitor microglia lines. Lens, ×20; scale bar, 100 μm.

Therefore, we next differentiated the isogenic *IL18RAP* 3′UTR lines into human microglia following the protocol of Haenseler et al.^67^ (Fig. 4a). iPSC-derived microglia differentiation was validated by immunofluorescence staining of the microglial-specific marker TMEM119 (Extended Data Fig. 9). In differentiated human microglia, we detected a ~five- to sixfold downregulation in the levels of the variant IL18RAP protein and in the levels of the *IL18RAP* mRNA relative to the canonical sequence of the isogenic line (Fig. 4b,c). Therefore, the variants at the 3′UTR regulate IL18RAP mRNA and protein expression and provide a conceivable explanation for the variant function in human C9-ALS microglia. Next, we investigated the molecular mechanism that controls the *IL18RAP* mRNA levels by performing an mRNA stability assay in human microglia. We measured an mRNA degradation rate that is twice as fast with the rare 3′UTR variants relative to the canonical sequence, after inhibition of mRNA transcription by actinomycin D (Fig. 4d). Thus, the mechanism for reduced *IL18RAP* mRNA levels is associated with the destabilization of *IL18RAP* mRNA via variants in the 3′UTR.

### Variant 3′UTR reduces binding of dsRNA-binding proteins

**Extended Data Fig. 10:**
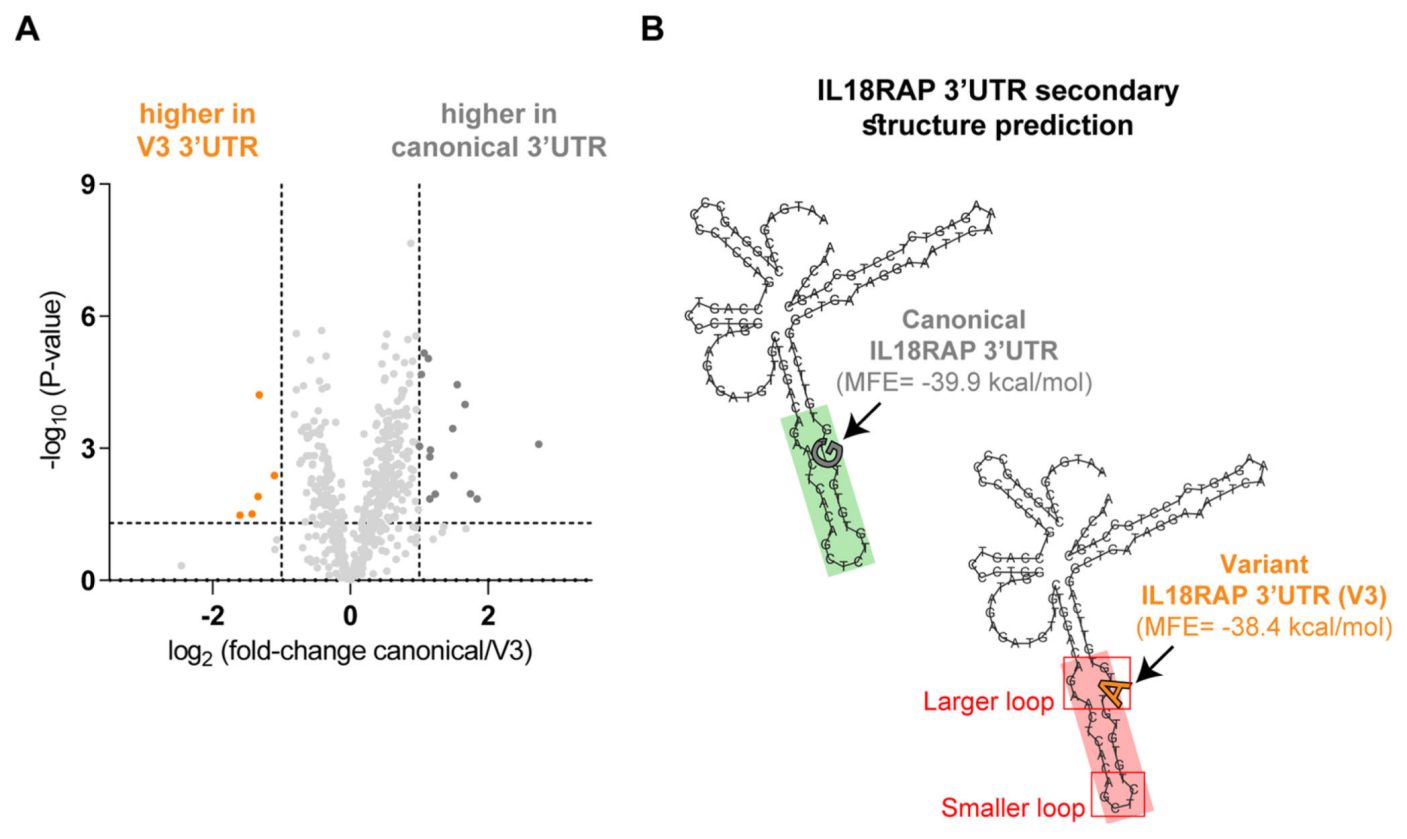
Differentially bound RNA binding proteins to variant 3′UTR (V3) relative to canonical 3′UTR. **(a)** Volcano plot of protein abundance associated with the canonical relative to variant (V3) IL18RAP 3′UTR (x-axis log2 scale), analyzed by MS. Y-axis depicts P-values (-log10 scale). Proteins significantly enriched in association with canonical/variant 3′UTR are colored (gray/orange). Features above the horizontal dashed line demarcate proteins with adjusted p < 0.05, in student’s t-test with FDR correction to multiple hypotheses. Vertical dashed lines are of 2 or ½ fold change (Supplementary Table 9). **(b)** Prediction of 3′UTR secondary structure by RNA Fold^90^, suggests a minor change to the structure of the sequence harboring a V3 variant (red), relative to the canonical 3′UTR (green).

**Fig. 5:**
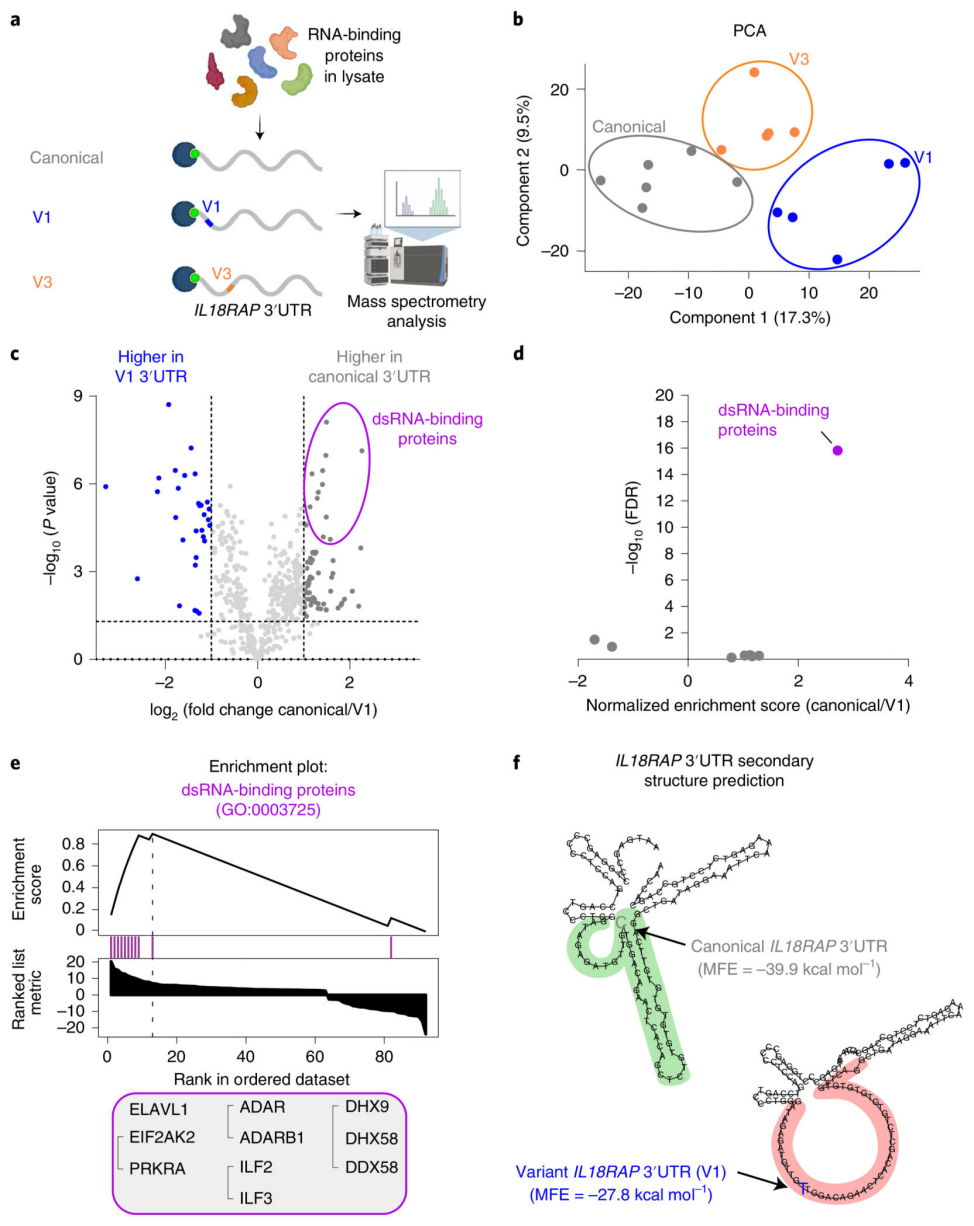
Reduced association of dsRNA-binding proteins to the variant *IL18RAP* 3′UTR. **a**, Diagram of mass spectrometry of RNA-binding proteins pulled down by *IL18RAP* 3′UTR sequences (canonical, V1 and V3). **b**, PCA of *IL18RAP* 3′UTR-associated proteomes pulled down by the canonical (gray; *N* = 6 experimental repeats), V3 (orange; *N* = 5) and V1 (blue; *N* = 5) biotin-tagged, in vitro transcribed oligonucleotides. **c**, Volcano plot of protein abundance associated with the canonical relative to variant (V1) *IL18RAP* 3′UTR (*x* axis, log_2_ scale) analyzed by mass spectrometry. The *y* axis depicts *P* values (–log_10_ scale). Proteins significantly enriched in association with canonical/variant 3′UTR are colored (gray/blue). dsRNA-binding proteins are demarcated by a purple oval. Features above the horizontal dashed line demarcate proteins with adjusted *P* values of <0.05 in a Student’s *t*-test with FDR correction for multiple hypotheses. Vertical dashed lines are of 2- or 0.5-fold change. A non-significant data point of KIF13B (*P* = 0.08) is not shown for clarity of the illustration (Supplementary Table 9). **d**, Volcano plot of normalized enrichment score of the Gene Ontology (GO) molecular function gene sets from GSEA analysis of differentially expressed proteins (canonical versus V1 *IL18RAP* 3′UTR). Reduced association of dsRNA-binding proteins (GO:0003725; purple) with V1 *IL18RAP* 3′UTR relative to the canonical 3′UTR. All gene sets are described in Supplementary Table 10. **e**, Profile of GSEA enrichment score and positions of the ten dsRNA-binding proteins (purple) within all differentially expressed proteins, ranked from most enriched in canonical 3′UTR to most depleted protein (Supplementary Table 10; WebGestalt^89^). **f**, Prediction of 3′UTR secondary structure by RNA Fold^90^ suggests a more stable dsRNA structure of canonical 3′UTR (green) with lower minimum free energy (MFE) than that of the sequence harboring a V1 variant (red).

We sought the potential *trans-acting* factors that might differentially bind to the canonical and variant 3′UTRs. To this end, we performed RNA pulldown assays and mass spectrometry on in vitro transcribed canonical and variant forms of the *IL18RAP* 3′UTRs V1 and V3 (Fig. 5a). Mass spectrometry after pulldown identified 552 proteins with good confidence (passed all quality control filters, found in 50% of the repeats in at least one experimental group and were represented by at least two unique peptides; Supplementary Table 9) that were enriched in comparison to the negative control. Principal-component analysis (PCA) demonstrated a clear separation of proteomes bound by the canonical and variant *IL18RAP* 3′UTRs (Fig. 5b and Supplementary Table 9). Gene set enrichment analysis (GSEA) revealed a reduction in the association of dsRNA-binding proteins to V1 *IL18RAP* 3′UTR relative to the canonical 3′UTR (ELAVL1/Hur, PRKRA, EIF2AK2/PKR, ADAR, ADARB1, ILF2, ILF3, DHX9, DHX58 and DDX58; Fig. 5c–e and Supplementary Table 10). These dsRNA-binding proteins were reported in other contexts to play roles in controlling the stability of mRNA^68–74^, consistent with the observed changes to *IL18RAP* mRNA stability. A similar analysis of the V3 variant was unproductive (Extended Data Fig. 10a).

In accordance, RNA Fold analysis predicted that the canonical 3′UTR sequence consists of a more stable dsRNA structure than the V1 variant sequence (minimum free energy of canonical and variant *IL18RAP* 3′UTRs, -39.9 kcal mol^−1^ and -27.8 kcal mol^−1^, respectively; Fig. 5f and Extended Data Fig. 10b). In light of these results, we propose that variant-dependent changes to the secondary structure of the *IL18RAP* 3′UTR attenuate the binding of one or more of the dsRNA-binding proteins and may be involved in controlling the stability of *IL18RAp* mRNA.

### Variant *IL18RAP* 3′UTR reduces microglia neurotoxicity

**Fig. 6:**
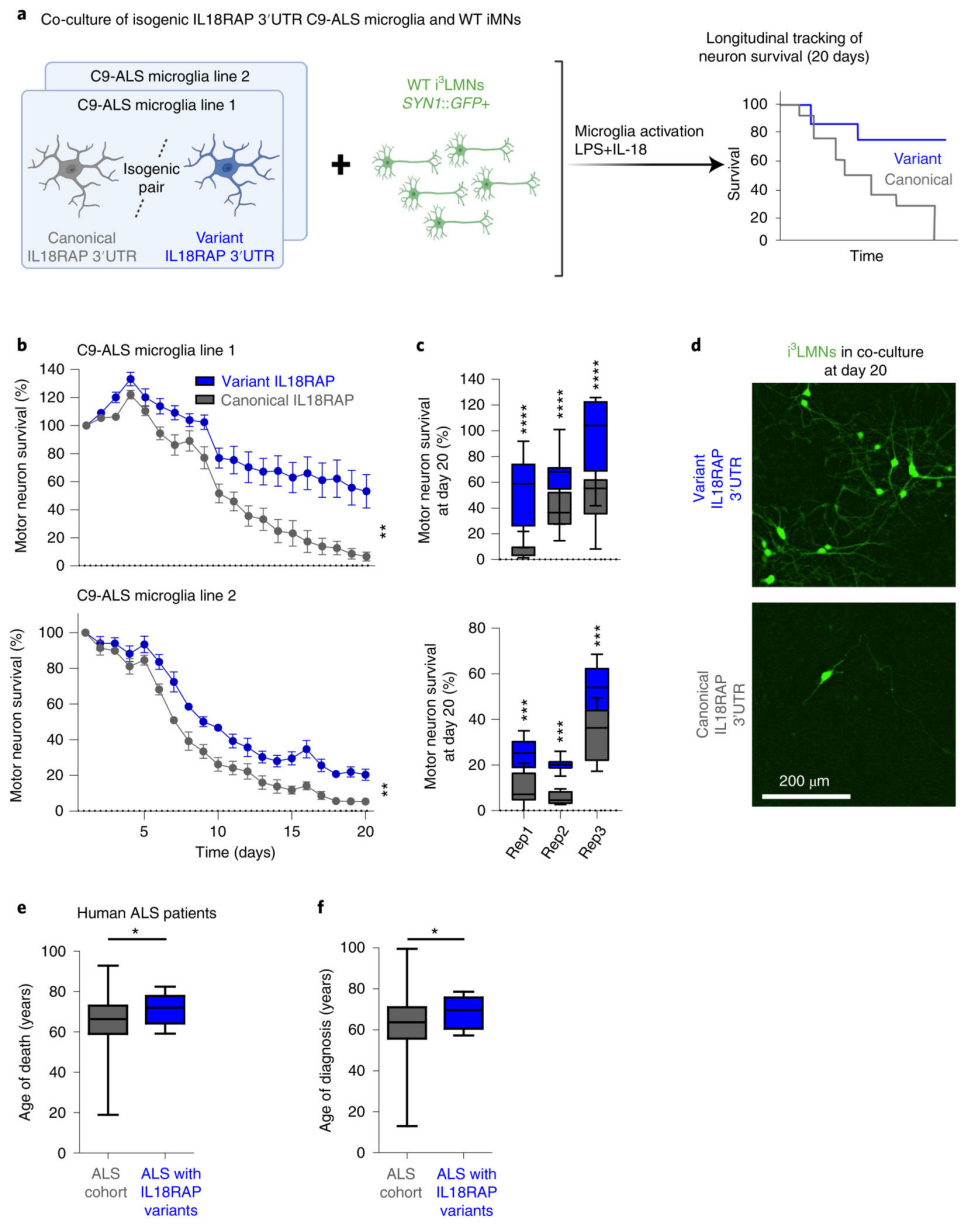
Variant *IL18RAP* 3′UTR is protective in human microglia and in individuals with ALS. **a**, Diagram of experimental design. Coculture of human i^3^LMNs that express green fluorescent protein (GFP), driven by the synapsin (*SYN1*) promoter (healthy, non-ALS^75^) and human iPSC-derived isogenic *IL18RAP* 3′UTR microglia (on a *C9orf72* repeat expansion background). Time-lapse microscopic analyses of i^3^LMN survival after microglia activation with a cocktail of LPS and the cytokine IL-18. **b**,**c**, i^3^LMN survival over 20 d in the presence of microglia harboring variant (blue) or canonical (gray) *IL18RAP* 3′UTR (two independent isogenic pairs based on independent human *C9orf72* lines; *n* = 3 independent differentiation procedures from different passages per line with three to eight coculture wells per passage). **b**, Survival plot of i^3^LMNs in a representative experiment for each isogenic pair (mean and s.e.m. values are shown; two-way ANOVA; C9-ALS microglia line 1, *P* = 0.006; C9-ALS microglia line 2, *P* = 0.0065). **c**, Box plot depicting the percentage of i^3^LMN survival on day 20 of coculture of all experiments. For box plots, the median is indicated by the central line, upper and lower quartiles are indicated by the box, and maximum/minimum values are indicated by the whiskers. Two independent isogenic pairs based on independent human *C9orf72* lines are shown; *n* = 3 independent differentiation procedures from different passages per line with three to eight coculture wells per passage (two-way ANOVA followed by Tukey’s multiple comparison test; C9-ALS microglia line 1, ****P < 0.0001; C9-ALS microglia line 2, *P* = 0.0006 for all repeats). **d**, Representative micrographs of fluorescent i^3^LMNs after 20 days of culture with C9-ALS microglia. **e**, Association of age of death (9 individuals with protective 3′UTR variants/4,263 individuals with ALS with available phenotypic data in Project MinE and NYGC cohorts). **f**, Association of age of diagnosis (8 individuals with protective 3′UTR variants/4,216 individuals with ALS with available phenotypic data in Project MinE and NYGC cohorts). The *IL18RAP* variant is associated with delayed age of death (+6.1 years; permutation *P* = 0.02; Cohen’s *d* effect size = 0.65) and age of diagnosis (+6.2 years; permutation *P* = 0.05, Cohen’s *d* effect size = 0.62) relative to the mean age of all Project MinE and NYGC individuals with ALS. For box plots, the median is indicated by the central line, upper and lower quartiles are indicated by the box, and maximum/minimum values are indicated by the whiskers; *P < 0.05; **P < 0.01; ***P < 0.001; ****P < 0.0001.

To study the potential protective impact of *IL18RAP* 3′UTR mutations, we performed survival analyses in a coculture system of human iPSC-derived isogenic *IL18RAP* 3′UTR microglia (on a *C9orf72* repeat expansion background) with human iPSC-derived lower motor neurons (i^3^LMNs; healthy, non-ALS^75^). Time-lapse microscopy was used to quantify motor neuron survival after microglia activation with a cocktail of lipopolysaccharide (LPS) and the cytokine IL-18 (Fig. 6a). Motor neuron survival was significantly improved in the presence of microglia harboring the *IL18RAP* 3′UTR variants relative to microglia harboring the canonical *IL18RAP* 3′UTR (two independent isogenic pairs based on independent human *C9orf72* lines; *n* = 3 independent differentiation procedures from different passages per line with three to eight coculture wells per passage; Fig. 6b–d and Supplementary Video 1). Based on these studies, we conclude that rare variants of the *IL18RAP* 3′UTR increase *C9orf72* microglia-dependent motor neuron survival and hence convey a protective property.

### Variant *IL18RAP* 3′UTR endows survival advantage to individuals with ALS

To determine whether the variants in the *IL18RAP* 3′UTR are also protective in individuals with ALS, we tested the association between age of diagnosis and age of death in individuals with ALS harboring canonical or variant forms of *IL18RAP* 3′UTR. Of 4,216 individuals for whom data on the age of diagnosis were available (Project MinE and NYGC cohorts), 8 harbored *IL18RAP* 3′UTR variants. Of 4,263 individuals for whom the age of death was available, 9 harbored *IL18RAP* 3′UTR variants. *IL18RAP* 3′UTR variants are expected to be depleted in ALS genomes; nonetheless, in those extremely rare individuals harboring *IL18RAP* 3′UTR variants, these were associated with an older age of death and an older age of diagnosis. On average, the age of death was higher by 6.1 years than that observed for individuals with canonical *IL18RAP* 3′UTR (permutation *P* = 0.02; Cohen’s *d* effect size = 0.65; Fig. 6e and Supplementary Table 11), and the age of diagnosis was higher by 6.2 years than that observed for individuals with canonical *IL18RAP* 3′UTR (permutation *P* = 0.05; Cohen’s *d* effect size = 0.62; Fig. 6f and Supplementary Table 11). Thus, variants in *IL18RAP* 3′UTR are protective against ALS in a tissue culture model and correlate with survival advantage for individuals suffering from the disease.

### Variant *IL18RAP* 3′UTR dampens NF-κB signaling in microglia

**Fig. 7:**
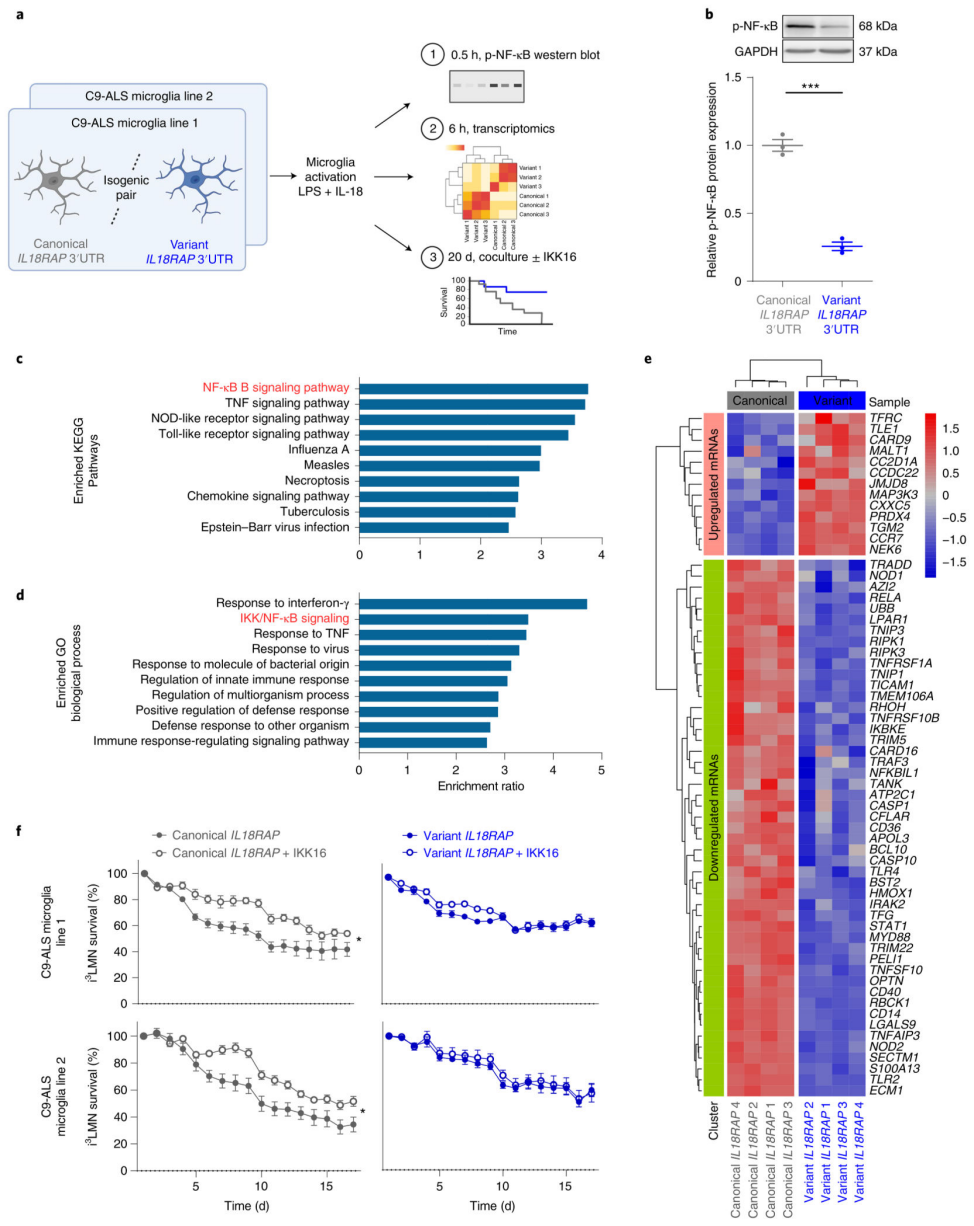
Variant *IL18RAP* 3′UTR dampens neurotoxic NF-κB signaling in human microglia. **a**, Diagram of experimental design. Four *IL18RAP* 3′UTR lines (two isogenic pairs) were differentiated into human microglia^67^ and analyzed for p-NF-κB protein levels, transcriptomics and neuronal survival in coculture with/without a selective IκB kinase (IKK) inhibitor (IKK16), following activation with a cocktail of LPS and the cytokine IL-18 for 0.5 h, 6 h and 20 d, respectively. **b**, Western blotting analysis revealed reduced levels of p-NF-κB in microglia with variant *IL18RAP* 3′UTR relative to isogenic control. Scatter dot plots show mean and s.e.m. values (two-sided *t*-test; *P* = 0.0002, *N* = 3 independent experiments). **c-e**, mRNA extracted from human microglia was subjected to a next-generation sequencing with downstream bioinformatics studies. ORA within KEGG Pathways (**c**) and GO biological processes (**d**) of the differentially expressed transcriptome in microglia harboring variant versus canonical *IL18RAP* 3′UTR. Bar graphs depicting the ratio of enrichment for significantly enriched pathways (FDR ≤ 0.05) are shown (Supplementary Table 13; WebGestalt^89^). An unsupervised study of the NF-κB transcriptomic signature (GO:0007249 pathway-associated mRNAs) in microglia with the variant (blue) relative to the isogenic canonical (gray) *IL18RAP* 3′UTR is shown (**e**). High expression is in red, and low expression is in blue. **f**, Time-lapse microscopic analyses of cocultured human i^3^LMNs (healthy, non-ALS^75^) with human iPSC-derived isogenic *IL18RAP* 3′UTR microglia (on a *C9orf72* repeat expansion background), activated with a cocktail of LPS and the cytokine IL-18, without (carrier alone; DMSO) or with IKK16 (200 nM), a selective IKK inhibitor that inhibits NF-κB signaling^80^. IKK16 significantly ameliorates motor neuron death relative to control only in the context of canonical *IL18RAP* 3′UTR, but did not further contribute to rescue in human microglia with the protective variant *IL18RAP* 3′UTR; two independent isogenic pairs based on independent human *C9orf72* lines with three to eight coculture wells per line (a survival plot with mean and s.e.m. values is shown; two-way ANOVA; C9-ALS microglia line 1, *P* = 0.01; C9-ALS microglia line 2, *P* = 0.025; *P < 0.05).

To study the role of NF-κB signaling in our system, we analyzed NF-κB phosphorylation and the impact on the transcriptome after microglia activation (Fig. 7a). Western blot analysis revealed reduced levels of p-NF-κB in variant *IL18RAP* 3′UTR relative to isogenic control (Fig. 7b). Reduced phosphorylation is associated with decreased nuclear localization and transcriptional activity of NF-κB^76–79^. In parallel, we conducted a next-generation sequencing study (Supplementary Table 12; Gene Expression Omnibus accession number GSE186757) of the differentially expressed transcriptomes in microglia harboring variant versus canonical *IL18RAP* 3′UTR. Overrepresentation analysis (ORA) of differentially expressed genes (DEGs) revealed downregulation of the NF-κB signaling pathway in microglia harboring the variant *IL18RAP* 3′UTR (KEGG Pathway enrichment results: ratio = 3.77, FDR *P* = 7.34 × 10^−6^; GO biological process enrichment results: ratio = 3.48, FDR *P* = 3.70 × 10^−12^; Fig. 7c,d and Supplementary Table 13). In addition, an unsupervised study of NF-κB pathway mRNAs (GO:0007249) demonstrated broad downregulation of pathway-associated mRNAs in microglia with the variant *IL18RAP* 3′UTR relative to the isogenic control (Fig. 7e). Therefore, the microglial NF-κB transcriptomic signature depends on signaling via the IL-18 receptor and is attenuated by protective *IL18RAP* 3′UTR variants.

To test a plausible neurotoxic role for NF-κB downstream of the IL-18R in this system, we next performed a coculture survival assay with or without IKK16, a selective IκB kinase (IKK) inhibitor that inhibits NF-κB signaling^80^. In human microglia with the canonical *IL18RAP* 3′UTR, IKK16 significantly ameliorated motor neuron toxicity relative to control (carrier alone; Fig. 7f). However, in human microglia with the protective variant *IL18RAP* 3′UTR, inhibition of NF-κB had no effect (two independent isogenic pairs based on independent human *C9orf72* lines with three to eight coculture wells per line; Fig. 7f). This suggests that NF-κB’s neurotoxic function resides epistatically downstream of IL18RAP in human microglia. Together, rare variants in the *IL18RAP* 3′UTR diminish NF-κB signaling, thus increasing *C9orf72* microglia-dependent motor neuron survival.

## Discussion

By performing rare variant aggregation analysis in regulatory noncoding regions on data from Project MinE and NYGC ALS sequencing consortia, we demonstrate that variants in the 3′UTR of *IL18RAP* are enriched in non-ALS genomes, indicating that these are relatively depleted in ALS. *IL18RAP* 3′UTR variants reduced the chance of developing ALS fivefold and delayed onset and therefore age of death in individuals with ALS.

Neuroinflammation is prevalent in neurodegeneration, including in ALS^44^, and is often characterized by the activation of microglia^30,43–48^. The cytokine IL-18 is part of this neuroinflammatory milieu, activating intracellular signaling cascades, including NF-κB.

We demonstrate the downregulation of variant *IL18RAP* 3′UTR, which might be particularly relevant because elevated levels of the cytokine IL-18 (refs. ^39–41^) and IL18RAP were measured in several forms of ALS. Mechanistically, we demonstrate destabilization of *IL18RAP* by the variant 3′UTR, which is consistent with the reduced propensity of the 3′UTR to form a double-stranded secondary structure and accordingly, reduced binding of dsRNA-binding proteins that are known to stabilize mRNAs. As a consequence, IL18RAP expression and NF-κB signaling are dampened in microglia.

We demonstrate the neuroprotective effect of the variant *IL18RAP* 3′UTR using CRISPR-edited human isogenic *C9orf72* microglia. Mechanistically, it is because IL18RAP is epistatically upstream of NF-κB in this system. Thus, the variant *IL18RAP* 3′UTR attenuates NF-κB activity and the expression of a broad set of NF-κB effector genes.

Our study resonates with the existence of protective variants in protein-coding regions in Alzheimer’s disease^81–84^ and in ALS^85,86^ and emphasizes the importance of seeking functional protective variants in association studies in neurodegeneration.

The discovery of a protective non-protein-coding allele is in line with previous reports of non-protein-coding variants in *VEGF* promoter/5′UTR, pre-miR-218-2 and *CAV1/CAV2* enhancers^9,87,88^, which were all deleterious. Together, these studies underscore the need to systematically explore the noncoding genome in brain diseases.

One limitation of our study is that the *IL18RAP* 3′UTR signal did not reach the conventional exome-wide multiplicity-adjusted significance threshold (*α* ≈ 2.6 × 10^−6^; ref. ^10^). However, the *IL18RAP* 3′UTR signal is comparable to that of protein-coding ALS-causing genes, such as *SOD1* and *NEK1*. Furthermore, the key findings were reproduced in a genome-wide study of all human 3′UTRs and in an independent replication study. Limitations in the statistical power might have prevented the discovery of other noncoding variants and may be overcome with larger ALS and control cohorts, which are not currently available. Additionally, we have focused our tissue culture studies on human *C9orf72* microglia. Therefore, the involvement of the *IL18RAP* 3′UTR in other ALS-associated genetic backgrounds remains to be experimentally explored, as is the relevance to other neurodegenerative diseases. Finally, the mechanism underlying IL18RAP dose sensitivity is not fully understood. While we provide evidence that the variant *IL18RAP* 3′UTR endows neuroprotection via dampening of microglia-dependent neurotoxicity, additional studies should explore the degree to which other cell types, such as motor neurons and astroglia, are involved.

In summary, we have identified the *IL18RAP* 3′UTR as a noncoding genetic disease modifier by rare variant association analysis of WGS data using ALS case–control cohorts. We show that IL-18 signaling modifies ALS susceptibility and progression, delineating a neuroprotective pathway and identifying potential therapeutic targets for ALS. Whereas the 3′UTR of *IL18RAP* is a protective noncoding allele associated with a neurodegenerative disease, the increasing wealth of WGS data in Project MinE, NYGC and elsewhere indicates that the exploration of noncoding regulatory genomic regions should reveal further disease-relevant genetic mechanisms.

## Methods

### Human genetic cohorts

All participants contributed DNA after signing informed consent at the submitting sites. Human materials were studied under approval of the Weizmann Institute of Science Institutional Review Board (Weizmann IRB 1039-1).

For the discovery cohort, Project MinE ALS sequencing consortium Datafreeze 1 includes 3,955 individuals with ALS and 1,819 age- and sex-matched healthy individuals free of any neurodegenerative disease, for a total of 5,774 quality control-passing whole genomes from the Netherlands, Belgium, Ireland, Spain, United Kingdom, United States and Turkey. Rare variant association in individuals with ALS versus healthy individuals was evaluated for regions of interest, when we could identify ≥two variants per region by SKAT-O, SKAT, CMC and VT in the RVTESTS environment^91^, with sex and the top 10 PCs as covariates. To construct the PCs of the population structure, an independent set of ~450,000 single-nucleotide polymorphisms (SNPs) was sampled from WGS data, (MAF ≥ 0.5%) followed by linkage disequilibrium (LD) pruning. Rare genetic variants were included based on a MAF of ≤ 0.01 within the healthy individuals in the current data set.

Replication cohorts were used for testing rare variant alleles (MAF < 0.01) in human *IL18RAP* 3′UTR (GRCh37/hg19 chr2:103068641–103069025 or GRCh38 chr2:102452181–102452565). The NYGC ALS Consortium (2,184 ALS Spectrum MND and 263 non-neurological control genomes from European/Americas ancestries), NHLBI’s TOPMed (62,784 non-ALS genomes) and gnomAD (5,537 non-ALS genomes; Europeans, non-Finnish, non-TOPMed) data were used. Joint analysis in the replication cohort was performed by chi-squared test with Yate’s correction. Meta-analysis was not possible because TOPMed and gnomAD covariate information is not available. From Project MinE Datafreeze 2, ~1,300 European heritage ALS genomes without Middle Eastern (Turkish and Israelis) genomes were used.

In general, sample size was measured based on collecting as many available ALS genomes and matched healthy controls. No statistical methods were used to predetermine sample sizes, but our sample sizes are similar to those reported in previous publications^9,62^. The human genetics study was a case–control cohort study; therefore, randomization of experimental groups and blinding was not relevant. All analyses for molecular biology studies were performed in a blinded manner.

### Quality control procedures in Project MinE genomics

Sample selection, data merging and sample- and variant-level quality control procedures for Project MinE ALS sequencing consortium genomes are described in full previously^62^. Briefly, 6,579 Project MinE ALS sequencing consortium whole genomes were sequenced on Illumina HiSeq2000 or HiSeqX platforms. Reads were aligned to the human genome build hg19, and sequence variants were called with the Isaac pipeline^92^. Individual genomic variant call format files (GVCFs) were merged with the ‘agg’ tool, a utility for aggregating Illumina-style GVCFs. Following completion of the raw data merge, multiple quality control filtering steps were performed: (1) setting genotypes with GQ < 10 to missing, (2) removing low-quality sites (QUAL < 30 and QUAL < 20 for SNPs and indels, respectively), (3) removing sites with missingness of > 10%, (4) samples were excluded if they deviated from the mean by more than 6 s.d. for total numbers of SNPs, singletons and indels, transition/transversion (Ti/Tv) ratio, heterozygous/homozygous-non-reference ratio and inbreeding (by cohort), (5) missingness > 5%, (6) genotyping–sequence concordance (made possible by genotyping data generated on the Illumina Omni 2.5M SNP array for all samples; 96% concordance), (7) depth of coverage, (8) a gender check (to identify mismatches), (9) relatedness (drop samples with >100 relatedness pairs), (10) related individuals were further excluded until no pair of samples had a kinship coefficient of >0.05 and (11) missing phenotype information. Following quality control, 312 samples with expended/inconsistent *C9orf72* status were omitted from further analysis. A total of 5,774 samples (3,955 individuals with ALS and 1,819 healthy individuals) passed all quality control measures and were included in downstream analysis. Per-nucleotide site quality control was performed on quality control-passing samples only; for biallelic sites: variants were excluded from analysis based on total depth (DP < 10,000 or >226,000), missingness of >5%, passing rate in the whole data set of <70%, sites out of Hardy–Weinberg equilibrium (by cohort, controls only, *P* < 1 × 10^−6^) and sites with extreme differential missingness between samples from individuals with ALS and healthy individuals (overall and by cohort, *P* < 1 × 10^−6^). Non-autosomal chromosomes and multiallelic variants were excluded from analysis.

### Selection of regions of interest

Discontinuous regions of interest approximating in total ~5 Mb include coding sequences and 3′UTRs of 295 genes (Supplementary Table 3) encoding proteins that were (1) previously reported to be associated with ALS, (2) RNA-binding proteins, including miRNA biogenesis or activity factors (University of California Santa Cruz gene annotation^93^), and (3) all 1,750 autosomal human pre-miRNA genes (miRBase v20 (ref. ^57^)). In addition, genome-wide analysis of all known human 3′UTRs (RefSeq^63^) was performed. Variants in regions of interest were extracted from Project MinE ALS sequencing consortium genomes using vcftools^94^ according to BED files containing genomic coordinates of interest (hg19) ±300 base pairs (bp) that ensure covering splice junctions and sequence (Supplementary Table 14).

### Annotation and region-based rare variant association analysis

After quality control and extraction of regions of interest, we performed functional annotation of all variants. Indels were left aligned and normalized using bcftools, and multiallelic sites were removed. For variant annotation, we developed a pipeline that calculates the impact of genetic variation in coding regions and in 3′UTR and miRNA regions using ANNOVAR^95^. The frequency of the variants in the general population was assessed by screening the 1000 Genomes Project, the Exome Aggregation Consortium and the NHLBI Exome Sequencing Project. For protein-coding open reading frames, association analysis of deleterious rare variants was performed, that is, frameshift variants, deviation from canonical splice variants, stop gain/loss variants or a non-synonymous substitution, as predicted by at least three prediction programs (SIFT, Polyphen2 HVAR, LRT, MutationTaster, MutationAssessor, FATHMM and MetaLR) in the dbNSFP environment (v2.0)^58^.

Noncoding sequence region-based rare variant association analysis included 3′UTRs and variants in miRNA recognition elements in 3′UTRs (Supplementary Table 3). Variants that impaired conserved miRNA-binding sites in 3′UTRs (predicted loss of function) were called by TargetScan (v7.0)^96^. Newly created miRNA-binding sites in 3′UTRs (predicted gain of function) were called by textual comparison of all possible mutated seeds around a variant to all known miRNA seed sequences in the genome, all human pre-miRNAs (mirBase v20)^57^ and miRNA:target gene networks, mature miRNA sequences (mirBase v20)^57^ and cognate targets within the 3′UTRs (Supplementary Table 3). Variant annotation scripts are available at GitHub at https://github.com/TsviyaOlender/Non-coding-Variants-in-ALS-genes-.

### Mammalian cell cultures

LCLs from the UK MNDA DNA Bank^64^ were originally derived from 16 different individuals: 4 healthy individuals (without ALS) carrying the canonical *IL18RAP* 3′UTR sequence (control; canonical *IL18RAP* 3′UTR), 4 individuals with sporadic ALS carrying the canonical *IL18RAP* 3′UTR sequence (sALS; canonical *IL18RAP* 3′UTR), 2 healthy individuals carrying a variant form of the *IL18RAP* 3′UTR (control; variant *IL18RAP* 3′UTR), 2 individuals with sporadic ALS carrying a variant form of the *IL18RAP* 3′UTR (sALS; variant *IL18RAP* 3′UTR) and 4 *C9orf72* individuals with ALS carrying the canonical *IL18RAP* 3′UTR sequence (*C9orf72;* canonical *IL18RAP* 3′UTR) (cell lines are listed in Supplementary Table 8; Weizmann IRB, 537-1). LCLs were cultured in RPMI1640 (Gibco, 21875091) with 20% inactivated fetal bovine serum (FBS; Biological Industries, 04-001-1A), 1% L-glutamine and 1% penicillin–streptomycin (Biological Industries, 03-0311B) at 37 °C and 5% CO2. Human bone osteosarcoma epithelial cells (U2OS; U-2 OS, ATCC HTB-96) were maintained in Dulbecco’s Modified Eagle Medium (DMEM; Biological Industries, 01-050-1A) supplemented with 10% FBS and 1% penicillin–streptomycin at 37 °C and 5% CO2. Human iPSCs were cultured on Matrigel-coated (Corning, 354277) plates in mTeSR1 medium (StemCell Technologies, 85850), according to the manufacturer’s instructions. Briefly, cells were passaged at 70–90% confluency with StemPro accutase (Gibco, A11105-01) and seeded in mTeSR1 medium supplemented with 10 nM Y-27632 dihydrochloride (Tocris, 1254). Cells were refreshed with mTeSR1 medium every 24 h until passage.

### Isolation and culture of rat cortical astrocytes

All experiments were performed in accordance with relevant guidelines and regulations of the Institutional Animal Care and Use Committee at Weizmann Institute of Science (IACUC 09491120-1). Primary cortical a**s**trocytes were isolated and cultured as previously described^97^ with several modifications. Briefly, the cerebral cortex of postnatal day 1 male Sprague–Dawley rat pups (purchased from Envigo) was dissected and placed in DMEM/F12 containing 0.5% trypsin (Biological Industries, 03-046-5B). After a 30-min incubation at 37 °C in a water bath, the cortical tissues were mechanically dissociated with a pipette into single cells and were seeded on poly-D-lysine-coated (Sigma-Aldrich, 7405) T75 culture flasks in astrocyte medium (DMEM/F12 (Gibco, 31330) supplemented with 10% FBS, 50 U ml^−1^ penicillin–streptomycin and 2 mM GlutaMAX (Gibco, 35050038)). The confluent cultures were shaken for 4 h at 200 r.p.m. to remove microglial cells. Each T75 flask was trypsinized and split into three new T75 flasks. After 7–8 d, the confluent flasks were trypsinized and frozen in 90% FBS and 10% DMSO until further use.

### I^3^LMN differentiation and *SYN*::GFP^+^ transduction

Differentiation of human iPSCs into LMNs (i^3^LMNs, iPSCs containing doxycycline-induced human NGN2, ISL1 and LHX3) was performed as described previously^75^. Briefly, iPSCs were seeded on day 0 into mTeSR1 medium supplemented with 10 nM Y-27632 dihydrochloride. A few hours after seeding, cells were transduced with *SYN*::GFP lentivirus (pHR-hSyn-EGFP; Addgene, 114215). Twenty-four hours after seeding the cells, medium was replaced with differentiation medium (DMEM/F12 (Gibco, 31330-038) with 1× MEM non-essential amino acids (Gibco, 11140-035), 2 mM GlutaMAX (Gibco, 35050038), 1× N-2 supplement (Gibco, 17502-048), 2 μg ml^–1^ doxycycline (Sigma-Aldrich, D9891-1G) and 10 nM Y-27632 dihydrochloride). On day 3, cells were split using accutase, counted and reseeded on poly-D-lysine-coated dishes containing rat astrocytes in neuronal medium (B27 electrophysiology medium (Gibco, A14137-01) supplemented with 1× MEM non-essential amino acids, 2 mM GlutaMAX, 1× N-2 supplement and 1 μg ml^−1^ mouse laminin (Gibco, 23017-015)). Twice a week, half of the medium was removed, and an equal volume of fresh medium was added.

### Generation of *IL18RAP* 3′UTR rare variant human iPSC lines

iPSCs were generated by the Ichida lab from human lymphocytes from individuals with ALS obtained from the NINDS Biorepository at the Coriell Institute for Medical Research. Lymphocytes were reprogrammed into iPSCs as previously described^65^. The NINDS Biorepository requires informed consent from individuals.

Human iPSC lines were maintained on irradiated MEFs in hESC medium (DMEM/F12 (Sigma-Aldrich, D6421) supplemented with 20% KO Serum Replacement (Gibco, 10828-028), 1% GlutaMAX (Gibco, 35050038), 1% MEM-NEAA (Biological Industries, 01-040-1A), 0.1 mM 2-mercaptoethanol (Gibco, 31350-010), 10 ng ml^−1^ hFGF (PeproTech, 100-18B)) and passaged twice a week with collagenase IV (Worthington, LS004188).

CRISPR guides were chosen using several design tools, including the MIT CRISPR design tool^98^ and sgRNA Designer, Rule set 2 (ref. ^99^), in the Benchling implementations (www.benchling.com) and SSC^100^ and sgRNAscorer^101^ in their websites.

Before the CRISPR procedure, iPSCs were passaged once under feeder-free conditions (LDEV Free GelTrex matrix (Gibco, A1413202) and mTESR1 medium (StemCell Technologies, 85850)), dislodged as single cells using StemPro Accutase (Gibco, A11105-01), washed twice with Opti-MEM (Gibco, 31985-047) and counted. Ninety microliters of cell suspension containing 1 million cells was mixed with 10 μl of DNA mix: 4 μg of pSpCas9(BB)-2A-Puro (PX459) plasmid (Addgene, 48139), 0.4 μg of guide RNA (gRNA)-encoding plasmid (pKLV-U6gRNA(BbsI)-PGKzeo2ABFP, derived from pKLV-U6gRNA(BbsI)-PGKpuro2ABFP (Addgene)), 1 μg (8 pmol) of ssODN repair template (Supplementary Table 15; IDT, 400 bases Megamer DNA Oligonucleotide) and 2.6 μg of carrier plasmid DNA. CRISPR reaction components were introduced to iPSCs by single-round electroporation using a Nepa21 system (NEPA GENE). One hundred microliters of cells and DNA suspension was transferred to a Nepa Electroporation Cuvette, 2-mm gap (Nepa Gene, EC-002). The following electroporation conditions were used: 150-V poring pulse, 5-ms pulse length, 20-V transfer pulse and 50-ms pulse length. Electroporated cells were transferred to two GelTrex-coated 100-mm dishes (1,000 and 10,000) in mTeSR medium supplemented with 10 μM ROCK inhibitor (PeproTech, 1293823) and placed into a CO_2_ incubator for 2 d. Forty-eight hours after electroporation, cells were treated with 0.5 μg ml^−1^ puromycin (Sigma-Aldrich) for 2 consecutive days. Cells that survived were maintained until clone development. Single clones were picked and transferred to 96-well plates. Matured clones were genotyped at the first passage. Additionally, the top five predicted off-target sites for the gRNA were sequenced (Supplementary Table 16). Selected clones containing desired mutations were expanded, cryopreserved and used for the downstream experiments.

### Differentiation and culturing of human iPSC-derived microglia

Human iPSCs were differentiated into microglia-like cells as previously described^67^. Briefly, to form embryoid bodies, iPSCs were seeded into 96-well suspension plates in mTeSR1 medium supplemented with 50 ng ml^−1^ rhBMP4 (Peprotech, 314-BP), 50 ng ml^−1^ VEGF (Peprotech, 100-20), 20 ng ml^−1^ SCF (Peprotech, 300-07) and 10 nM Y-27632 dihydrochloride. Every day, half of the medium was removed, and an equal volume of fresh medium was added. After 4 d, 12 embryoid bodies were transferred into each well of a six-well plate in X-VIVO 15 (Lonza, BE02-060Q) containing 100 ng ml^−1^ M-CSF (Peprotech, 300-25), 25 ng ml^−1^ IL-3 (Peprotech, 200-03), 2 mM GlutaMAX, 55 μM 2-mercaptoethanol (Gibco, 31350-10) and 100 U ml^−1^ penicillin–streptomycin (Biological Industries, 03-031-1B). iPSC-derived progenitor microglia (ipMGs) were collected weekly from the supernatant and were cocultured with iPSC-derived neurons in 96-well plates (Greiner, 655090) in neuronal medium containing 10 ng ml^−1^ IL-34 (Peprotech,200-34). Embryoid body medium was refreshed weekly.

### Immunofluorescence staining

ipMGs were fixed with 4% paraformaldehyde in PBS for 10 min, permeabilized with 0.2% Triton X-100 in PBS and blocked with CAS-Block (Life Technologies, 008130) for 10 min at room temperature and incubated with primary antibody (TMEM119 polyclonal antibody, Invitrogen, PA5-62505; 1:100) in CAS-Block overnight. Cells were washed three times with PBS for 5 min and incubated with secondary antibody (Cy2 AffiniPure donkey anti-rabbit IgG, Jackson Immunoresearch, 711-226-152; 1:200) in CAS-Block for 1 h at room temperature. After washing three times with PBS for 5 min each, cells were stained with DAPI, washed once with PBS, overlaid with PBS and imaged with an Olympus IX83-based Live-Imaging system equipped with a VisiScope CSU-W1 spinning disk.

### i^3^LMN survival assay

The survival assay was conducted by monitoring enhanced GFP (eGFP) signal on day 5 i^3^LMNs cocultured with two independent CRISPR-edited isogenic iPSC-derived microglia (harboring canonical or variant *IL18RAP* 3′UTRs) with a *C9orf72* genetic background. Cells were monitored for over 20 d using an Incucyte Live-Cell Analysis System (Sartorius). Daily longitudinal microscopic tracking was performed following LPS (100 ng ml^−1^) and IL-18 (100 ng ml^−1^) treatment. The i^3^LMN survival assay was performed using three individual replicates for each line, with three to eight coculture wells per condition. Twice a week, half of the medium was removed, and an equal volume of fresh medium containing LPS and IL-18 was added.

### Cloning

Full *IL18RAP* coding sequence (CDS) and 3′UTR sequence (2,223 bp) in pMX vector was purchased from GeneArt (Invitrogen; Supplementary Table 15) and subcloned with V5 epitope into pcDNA3. Different mutants, including wild-type *IL18RAP* CDS + mutant 3′UTR (V1 or V3) and a dominant-negative coding mutant E210A-Y212A-Y214A CDS + wild-type 3′UTR (3CDS)^31^, were created by transfer-PCR mutagenesis^102^. Next, wild-type and mutant full *IL18RAP* were subcloned into pUltra vector (a gift from M. Moore laboratory at Memorial Sloan-Kettering Cancer Center, New York, New York, United States of America; Addgene, 24130), for which mCherry was replaced with eGFP downstream of the human ubiquitin C promoter and EGFP-P2A. Cloning procedures were done via restriction-free cloning^103^. A list of primers used for cloning and transfer-PCR mutagenesis are described in Supplementary Table 16.

### Transfection

Transfection to U2OS cells in 1.9-cm^2^ corning plates was performed at 70–80% confluency 24 h after plating in antibiotic-free medium using Lipofectamine 2000 (0.5 μl per well; Thermo Fisher Scientific, 11668027). Each well was considered as a single replicate. For the NF-κB reporter assay, U2OS cells were induced with/without recombinant IL-18 (5 ng ml^−1^) 72 h after transfection with the full coding sequence of the *IL18RAP* coding region + 3′UTRs (pUltra vector 500 ng per 1.9-cm^2^ plate), luc2P/NF-κB-RE (pGL4.32, 100 ng) luciferase and *Renilla* luciferase (hRluc, 10 ng). After 6 h, cells were collected for the Dual-Luciferase Reporter Assay (E1960), and luminescence was quantified using a Veritas Microplate Luminometer.

### RNA extraction, cDNA synthesis and qPCR

Total RNA from LCLs was extracted using a Direct-Zol RNA MiniPrep kit (Zymo Research, R2052) according to the manufacturer’s instructions. Total RNA from ipMGs was extracted using an miRNeasy micro kit (QIAGEN, 217084) according to the manufacturer’s instructions. Total RNA was reverse transcribed using a High Capacity cDNA Reverse Transcription kit (Applied Biosystems, 4368814), according to manufacturer’s instructions, except for the mRNA stability assay, where an equal volume of RNA (and not equal amounts of RNA) from each sample was used to generate cDNA. qPCR was performed using TaqMan Universal PCR master mix (Applied Biosystems, 4304437) or KAPA SYBR FAST (Roche, KK4605). Primers and TaqMan probes are shown in Supplementary Table 16.

### Bulk MARS-seq

Two hundred thousand ipMGs harboring variant or canonical *IL18RAP* 3′UTRs (*n* = 4) were treated with 100 ng ml^−1^ LPS and 100 ng ml^−1^ IL-18 for 6 h in ipMG medium (Advanced DMEM (Gibco, 12491-015) containing 1× N-2 supplement (Gibco, 17502-048), 2 mM GlutaMAX (Gibco, 35050038), 55 μM 2-mercaptoethanol (Gibco, 31350-10), 50 U ml^−1^ penicillin–streptomycin (Biological Industries, 03-031-1B) and 100 ng ml^−1^ IL-34 (Peprotech, 200-34). After 6 h, RNA was extracted as described above, and a bulk adaptation of the MARS-seq protocol^104,105^ was used to generate 3′ RNA-seq libraries for expression profiling. Briefly, 50 ng of input RNA from each sample was barcoded during reverse transcription and pooled. Following Agencourt Ampure XP bead cleanup (Beckman Coulter), the pooled samples underwent second strand synthesis and were linearly amplified by T7 in vitro transcription. The resulting RNA was fragmented and converted into a sequencing-ready library by tagging the samples with Illumina sequences during ligation, reverse transcription and PCR. Libraries were quantified by Qubit and TapeStation as well as by qPCR for *GAPDH* gene expression as previously described^104,105^. Sequencing was done on a NovaSeq 6000 system with an SP Reagent kit for 100 cycles (Illumina; paired-end sequencing).

Analysis of the MARS-seq data was done using the UTAP pipeline^106^ (the Weizmann Institute Bioinformatics Unit) to map the reads to the human genome and to calculate unique molecular identifier counts per gene. Reads were trimmed from their adapter using cutadapt (parameters: -a AGATCGGAAGAGCACACGTCTGAACTCCAGTCAC -a ‘A(10)’ –times 2 -u 3 -u - 3 -q 20 -m 25) and mapped to the hg38 genome (STAR v2.4.2a). The pipeline removes unique molecular identifier redundancy and quantifies the 3′ of RefSeq annotated genes (1,000 bases upstream and 100 bases downstream of the 3′ end). Genes having a minimum of five reads in at least one sample were considered for further analysis. DEG detection and count normalization analyses were performed by DESeq2. *P* values in the UTAP results were adjusted for multiple testing using the Benjamini and Hochberg procedure. The following were used as thresholds for significant DEGs: adjusted *P* value of <0.01, | log2 fold change | ≥ 0.585 and baseMean > 20. This assay was done with critical advice from H. Keren-Shaul from the Genomics Sandbox unit at the Life Science Core Facility of Weizmann Institute of Science.

### Cell lysis and western blotting

LCLs were washed in 1× PBS, centrifuged at 800*g* for 5 min at 4 °C, pelleted and lysed in ice-cold RIPA buffer (Supplementary Table 17) supplemented with cOmplete Protease Inhibitor Cocktail (Roche, 4693116001) and PhosSTOP (Roche, 4906837001). The lysates were cleared by centrifugation at 15,000*g* for 10 min at 4 °C. Protein concentrations were quantified with Protein Assay Dye Reagent (Bio-Rad, 500-0006), and protein was resolved at 30–50 μg of total protein per well by 8–10% SDS–PAGE at 100–120 V for 70 min. After gel electrophoresis, proteins were transferred to nitrocellulose membranes (Whatmann, 10401383) at 250 mA for 70 min. Membranes were stained with Ponceau (Sigma, P7170), blocked for 1 h at room temperature with 3% bovine albumin fraction V (MPBio, 160069) or 5% milk protein in PBS containing 0.05% Tween-20 (PBST) and incubated with primary antibodies (Supplementary Table 18) overnight at 4 °C with rocking in antibody solution (5% albumin, 0.02% sodium azide and five drops of phenol red in 0.05% PBST). Following primary antibody incubation, membranes were washed three times for 5 min at room temperature with 0.05% PBST and incubated for 1 h at room temperature with horseradish peroxidase-conjugated species-specific secondary antibodies, washed three times for 5 min each in 0.05% PBST at room temperature and visualized using EZ-ECL Chemiluminescence (Biological Industries, 20500-120) by ImageQuant LAS 4000 (GE Healthcare Life Sciences). Densitometric analysis was performed using ImageJ v ij-1.52n (NIH).

### In vitro transcription of biotinylated *IL18RAP* 3′UTR

To identify the potential *trans*-acting factors that might differentially bind to the canonical and variant 3′UTRs, an RNA pulldown and mass spectrometry assay was performed on in vitro transcribed canonical and variant forms of the *IL18RAP* 3′UTRs, V1 and V3. Briefly, The canonical, V1 and V3 biotinylated *IL18RAP* 3′UTR sequences (384 nucleotides) and the negative control (ultrapure water only) were produced by using an in vitro transcription HiScribe T7 ARCA kit (NEB, E2060S) following the manufacturer’s instructions. Briefly, 300 ng of purchased DNA template (50 ng μl^−1^; Twist; Supplementary Table 15) was incubated with unlabeled ATP/GTP/CTP and 5% biotin-labelled UTP at 37 °C for 3 h. Next, DNase treatment was performed by incubating the reactions at 37 °C for 30 min and was followed by incubation at 65 °C for 10 min to terminate the reaction. The RNA products were purified by an RNA cleanup purification kit (Zymo Research, R1015). The concentrations of the purified RNA samples were measured by Nanodrop, and the expected length was analyzed by TapeStation.

### Pulldown of *IL18RAP* 3′UTR RNA-associated proteins

LCL pellets were suspended and lysed in RIPA buffer followed by centrifugation at 15,000*g* for 10 min at 4 °C. The concentrations of the cleared supernatants were measured by Bradford assay. One milligram of lysate per sample was incubated with Pierce streptavidin magnetic beads (Thermo Scientific, 88817) for 30 min at 4 °C in rotation to preclear the lysates from endogenous biotinylated proteins. To bind in vitro transcribed products (wild-type, V1, V3 and negative control; *n* = 6 repeats per group) to the beads, new prepared binding Pierce streptavidin magnetic beads were incubated by rotation with equal amounts of in vitro transcribed products for 30 min at 4 °C (100 μl of beads per 10 pmol of RNA product). After 30 min, the tubes of incubated in vitro transcribed products with beads were washed three times, and the cleared lysate was added equally to each tube and incubated for 30 min at 4 °C. In the next step, the samples were washed three times by magnetizing the beads and resuspended by vortexing with a high-salt buffer. The bound beads were magnetized and suspended in 20 μl of RNase-free 1× PBS for the on-bead digestion procedure.

### Liquid chromatography and mass spectrometry

The resulting peptides were analyzed using nanoflow liquid chromatography (nanoAcquity) coupled to high-resolution, high mass accuracy mass spectrometry (Q-Exactive HF). Each sample was analyzed on the instrument separately in a random order in discovery mode.

### Raw proteomic data processing

Raw mass spectrometry data were processed using MaxQuant version 1.6.6.0 (ref. ^107^). A database search was performed with the Andromeda search engine^108,109^ using the human UniProt database appended with common lab protein contaminants. A forward/decoy approach was used to determine the FDR and filter the data with a threshold of 1% FDR for both the peptide–spectrum matches and the protein levels. The label-free quantification algorithm in MaxQuant^110^ was used to compare between experimental samples. Additional settings included the following modifications: fixed modification, cysteine carbamidomethylation; variable modifications, methionine oxidation, asparagine and glutamine deamidation and protein N-terminal acetylation.

### Proteomics statistical analysis

The ProteinGroups output table was imported from MaxQuant to the Perseus v.1.6.2.3 environment^111^. Quality control excluded reverse proteins, proteins identified only based on a modified peptide and contaminants. Non-specific streptavidin bead binders were excluded by the following procedure: label-free quantification intensity values were log_2_ transformed, and two outlier samples were excluded from further analysis. Missing values were imputed by creating an artificial normal distribution with a downshift of 1.8 s.d. and a width of 0.4 of the original ratio distributions. A Student’s *t*-test with s0 = 0.1 was performed with an FDR *P* value of ≤0.05 between the experimental groups (canonical, V1 and V3) and the negative control group, which was defined as a single control group. Proteins that passed all quality control filters were separated for each of the experimental groups and compared to the negative control samples (ultrapure water). The statistically significantly associated proteins were filtered to retain only proteins that were found in 50% of the repeats in at least one experimental group and were represented by at least one unique peptide. The enriched proteins were subjected to Student’s *t*-test between every two groups (canonical versus V1 and canonical versus V3), with *S*_0_ = 0.1, FDR *P* ≤ 0.05 and a fold change threshold of >2.

### Processing of mouse brain samples for flow cytometry

Male C57BL/6J wild-type mice (~130 d old; *n* = 5 per experiment) were used for all mouse experiments unless otherwise specified. Mice were housed in groups, with a maximum of six mice per cage, in a passive air flow, environmentally ventilated cage system and maintained under specific pathogen-free conditions at the Walter and Eliza Hall Institute Animal Facility. Mouse cages were housed in an environmentally controlled room that was maintained at 20–21 °C with 60% relative humidity and a 14-h light/10-h dark cycle (6:00–20:00) with no external or natural light sources. Mice were killed with CO_2_ and perfused with PBS through the left ventricle of the heart. Dissected mouse cortex was cut into smaller pieces using scissors and digested in 0.5 mg ml^−1^ collagenase IV (Worthington Biochemical), 10 μg of deoxyribonuclease (Sigma-Aldrich) and 10% HI-FBS, RPMI1640 (Gibco) at 37 °C for 30 min with continuous agitation. Digested samples were gently triturated for 1 min, and the enzymatic reaction was stopped by adding 1 mM EDTA in PBS. The homogenate was filtered through a 100-μm cell strainer and centrifuged at 400*g* for 8 min at 4 °C to pellet the cells and myelin. This was followed by a myelin removal step by gradient centrifugation with 30% Percoll (Sigma-Aldrich) in PBS (700*g* for 20 min at 21 °C without brakes during deceleration). After myelin (the top white layer) separation, the middle transparent layer was collected, washed in PBS and centrifuged at 400*g* for 8 min at 4 °C to pellet the cells.

Cells pellets were incubated with Mouse Fc block (BD Biosciences, 553142), Fixable Viability Stain 620 (BD Biosciences, 564996) and the following antibody mixture in PBS at 4 °C for 30 min: BV421 rat anti-CD11b (BD Biosciences, 562605; 1:200; clone M1/70), BV510 hamster anti-mouse TCR β-chain (BD Biosciences, 563221; 1:200; clone H57-597), BV711 rat anti-mouse Ly-6G (BD Biosciences, 563979; 1:200; clone 1A8), APC-Cy7 rat anti-mouse CD45 (BD Biosciences, 557659; 1:200; clone 30-F11) and polyclonal goat IgG anti-mouse IL-18Rβ (R&D Systems, AF199; 1:200). Samples were then washed with PBS and incubated with Alexa Fluor 647 donkey anti-goat IgG (H + L) cross-adsorbed secondary antibody (Invitrogen, A-21447; 1:1,000) in PBS at 4 °C for 30 min. Surface-stained samples were washed with PBS and fixed and permeabilized with BD Fixation/Permeabilization solution (BD Biosciences, 554714) at 4 °C for 30 min, followed by intracellular staining with Alexa Fluor 488 anti-NeuN (EMD Millipore, MAB377X; 1:200; clone A60) and eFluor 570 anti-GFAP (eBioscience, 41-9892-82; 1:200; clone GA5) in BD Perm/Wash Buffer (BD Biosciences, 554714) at 4 °C for 30 min. Cells were washed with BD Perm/Wash Buffer and resuspended in PBS for analysis with a FACSymphony (BD Biosciences). Data were collected as FCS files and analyzed with FlowJo v10 software (BD Biosciences). Antibody specificity was assessed using relevant isotype control antibodies and fluorescence minus one. Compensation was adjusted using single-stained samples.

The expression of IL18RAP (IL-18Rβ) was expressed as mean fluorescence intensity or percent frequency after gating for the following cell types: immune cells (CD45^hi^), microglia (CD45^int^CD11^hi^), neurons (CD45^−^CD11b^−^NeuN^+^) and astrocytes (CD45^−^CD11b^−^GFAP^+^). Experimental use was in accordance with the Australian Code of Practice for the Care and Use of Animals for Scientific Purposes (Australian National Health and Medical Research Council) and approved by the Walter and Eliza Hall Institute Animal Ethics Committee (ethics application 2020.017).

### Statistical analysis

Statistics were performed with Prism Origin (GraphPad). A Shapiro–Wilk test was used to assess normality of the data. Pairwise comparisons passing normality testing were analyzed with a Student’s *t*-test, whereas the Mann-Whitney test was used for pairwise comparison of non-parametric data. Multiple group comparisons were analyzed using ANOVA with post hoc tests. For age of diagnosis and age of death, a permutation test was used (a Monte Carlo simulation test on the *t*-test between individuals with ALS harboring the canonical *IL18RAP* 3′UTR or variants of the *IL18RAP* 3′UTR). Statistical *P* values of <0.05 were considered significant. Data are shown as scatter dot plots with mean and s.e.m., box plots or as noted in the text.

### Reporting Summary

Further information on research design is available in the Nature Research Reporting Summary linked to this article.

## Extended Data

## Supplementary Material

Supplementary tables

Supplementary video

## Data Availability

Human genetics data are publically available from the sequencing consortia that control ethically appropriate usage of data, harmonization across studies and the safety of personal information donated by individuals that contributed their DNA for sequencing: the Project Mine ALS sequencing consortium, the NYGC ALS Consortium, the gnomAD and NHLBI’s TOPMed. Sequencing data are deposited at Gene Expression Omnibus under accession number GSE186757. All Other data used for this manuscript are available in the manuscript. The University of California Santa Cruz gene annotation^93^, miRBase v20 (ref. ^57^), RefSeq^63^, dbNSFP v2.0 (ref. ^58^) and ANNOVAR^95^ databases were used in this study. Source data are provided with this paper.

## Code availability

Variant annotation scripts are available at GitHub at https://github.com/TsviyaOlender/Non-coding-Variants-in-ALS-genes-.
